# Decreased cold‐inducible RNA‐binding protein (CIRP) binding to GluRl on neuronal membranes mediates memory impairment resulting from prolonged hypobaric hypoxia exposure

**DOI:** 10.1111/cns.70059

**Published:** 2024-09-24

**Authors:** Hui Jiang, Chenyan Lu, Haoyang Wu, Jie Ding, Jiayan Li, Jianfeng Ding, Yuqi Gao, Guohua Wang, Qianqian Luo

**Affiliations:** ^1^ Department of Hypoxic Biomedicine Institute of Special Environmental Medicine and Co‐innovation Center of Neuroregeneration, Nantong University Nantong China; ^2^ College of High‐Altitude Military Medicine Institute of Medicine and Hygienic Equipment for High Altitude Region, Army Medical University Chongqing China; ^3^ Key Laboratory of Extreme Environmental Medicine and High‐Altitude Medicine, Ministry of Education of China Chongqing China

**Keywords:** cold‐inducible RNA‐binding protein, glutamate receptor 1, high altitude, hypobaric hypoxia, learning and memory, TAT peptide

## Abstract

**Aim:**

To investigate the molecular mechanisms underlying memory impairment induced by high‐altitude (HA) hypoxia, specifically focusing on the role of cold‐inducible RNA‐binding protein (CIRP) in regulating the AMPA receptor subunit GluR1 and its potential as a therapeutic target.

**Methods:**

A mouse model was exposed to 14 days of hypobaric hypoxia (HH), simulating conditions at an altitude of 6000 m. Behavioral tests were conducted to evaluate memory function. The expression, distribution, and interaction of CIRP with GluR1 in neuronal cells were analyzed. The binding of CIRP to *GluR1* mRNA and its impact on GluR1 protein expression were examined. Additionally, the role of CIRP in GluR1 regulation was assessed using *Cirp* knockout mice. The efficacy of the Tat‐C16 peptide, which consists of the Tat sequence combined with the CIRP 110‐125 amino acid sequence, was also tested for its ability to mitigate HH‐induced memory decline.

**Results:**

CIRP was primarily localized in neurons, with its expression significantly reduced following HH exposure. This reduction was associated with decreased GluR1 protein expression on the cell membrane and increased localization in the cytoplasm. The interaction between CIRP and GluR1 was diminished under HH conditions, leading to reduced GluR1 stability on the cell membrane and increased cytoplasmic relocation. These changes resulted in a decreased number of synapses and dendritic spines, impairing learning and memory functions. Administration of the Tat‐C16 peptide effectively ameliorated these impairments by modulating GluR1 expression and distribution in HH‐exposed mice.

**Conclusion:**

CIRP plays a critical role in maintaining synaptic integrity under hypoxic conditions by regulating GluR1 expression and distribution. The Tat‐C16 peptide shows promise as a therapeutic strategy for alleviating cognitive decline associated with HA hypoxia.

## Graphical abstract

Exposure to high‐altitude hypoxia impairs memory by reducing CIRP and GluR1 levels in neurons, affecting synapse integrity. Tat‐C16 peptide administration ameliorates these impairments, suggesting a potential therapeutic strategy.

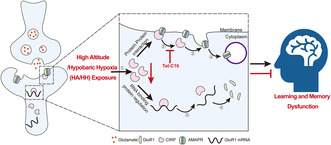

## 1 INTRODUCTION

Exposure to high altitudes (HAs) significantly impacts human physiology, particularly affecting cerebral oxygenation and, consequently, brain function.^1^, ^2^, ^3^ This phenomenon, primarily attributed to hypobaric hypoxia (HH), manifests as a reduction in cerebral tissue oxygenation, correlating directly with impaired cognitive abilities.^1^, ^4^ The severity of these impairments is closely linked to both the altitude and the duration of exposure.^4^ As individuals ascend to higher elevations, the reduced atmospheric pressure leads to decreased oxygen availability, challenging the body's ability to maintain optimal cerebral oxygenation levels.^2^, ^3^, ^4^ This condition triggers a cascade of physiological responses aimed at compensating for the reduced oxygen supply. However, over prolonged periods, it may lead to cognitive deficits due to increased intracranial pressure and hindered cerebral blood flow.^5^, ^6^ These changes not only exacerbate disorders in brain energy metabolism but also disrupt the normal excitatory or inhibitory conduction of neurons, thereby interfering with essential neural activities.^7^, ^8^, ^9^ Despite the recognition of HH‐induced cognitive deficits, the specific mechanisms underlying memory impairments at HA remain incompletely understood, underscoring the necessity of this research.

At the cellular level, synaptic plasticity, which includes processes such as long‐term depression (LTD) and potentiation (LTP), plays a fundamental role in learning and memory.^10^, ^11^ Synaptic plasticity refers to the ability of synapses to strengthen or weaken over time, in response to increases or decreases in their activity.^10^ LTD and LTP are considered cellular correlates of learning and memory, representing synaptic decrement and enhancement, respectively.^12^ Studies have shown that exposure to HH can significantly impact synaptic plasticity, thereby leading to various cognitive dysfunctions.^6^, ^9^, ^13^ The AMPA receptors (AMPARs), particularly the GluR1 subunit, are critical to this process, and these receptors are tetrameric or pentameric complexes that facilitate excitatory synaptic transmission and plasticity.^14^, ^15^, ^16^ The GluR1 subunit, abundantly expressed in the hippocampus and neocortex, plays a pivotal role in regulating synaptic strength, either through its expression, phosphorylation, or by mediating calcium influx, which are all underlying mechanisms of synaptic plasticity.^13^, ^14^, ^17^, ^18^, ^19^ However, the specific effects of HH on the expression and distribution of GluR1, and the consequent impact on learning and memory, remain areas of active investigation.

Another significant aspect of this study focuses on the cold‐inducible RNA binding protein (CIRP), a stress‐responsive factor upregulated in response to environmental stresses such as low temperature and hypoxia.^20^, ^21^ CIRP functions both extracellularly, as a novel member of the danger‐associated molecular patterns (DAMPs) family, and intracellularly, as an RNA‐binding protein that regulates the expression and distribution of proteins.^22^ Notably, CIRP interacts with other proteins, such as MD2, influencing the regulation of α5GABAA receptors.^23^, ^24^, ^25^ Research on the subject has begun to shed light on the complex interplay between CIRP, GluR1, and memory function under stress. However, while these studies provide valuable insights, they also reveal gaps in our understanding of how these proteins interact within the hypoxic stress response pathway to influence memory specifically in HA environments.

This study aims to bridge these gaps by exploring the roles of CIRP and GluR1 in memory impairment induced by HH. By elucidating the molecular mechanisms through which hypoxia affects these proteins and, consequently, cognitive function, this research seeks to contribute to the development of strategies for mitigating memory impairments in HA environments and other hypoxic conditions. Employing a specific mouse model subjected to conditions simulating a 6000 m HA exposure,^26^ our findings reveal that continuous HH exposure for 14 days leads to memory impairment. This exposure also results in a decrease in CIRP expression in the cytoplasm and a reduction of GluR1 expression in synaptosomes and neuronal membranes, with a concurrent increase in GluR1 levels within the neuronal cytoplasm. The reduction in CIRP destabilizes the distribution of GluR1 across neuronal membranes, a detrimental effect that can be mitigated by administering Tat‐C16 during HH exposure. These insights contribute to our understanding of the molecular pathways affected by HA exposure and offer potential targets for therapeutic intervention to mitigate cognitive impairments associated with such environments. The expected results also promise to contribute novel findings to the field, potentially guiding future research directions and therapeutic interventions for hypoxia‐related cognitive impairments.

## 2 MATERIALS AND METHODS

### 2.1 Animal experimentation

Male C57BL/6 mice, aged 8 weeks, were sourced from the Nantong University Experimental Animal Center. *Cirp* knockout mice were purchased from Cyagen Bioscience Inc. The mice were housed in stainless steel cages at a controlled temperature of 23 ± 1°C, under a 12 h light–dark cycle, with ad libitum access to food and water. Based on their body weights, the mice were randomly assigned to one of three groups (nine mice per group): the HH exposure for 14 days group (HH), the HH exposure for 14 days with peptide treatment group (HH + Tat‐C16), and the normobaric normoxia (NN) control group for the same duration. A 5 days acclimatization period was allowed before the commencement of experiments. As previously described,^27^ the HH and HH + Tat‐C16 groups were subjected to simulated altitude conditions equivalent to 6000 m for a duration of 14 days using a HH chamber (Shanghai Tawang, ProOx‐810), whereas the NN group was maintained under normobaric conditions.

We synthesized three distinct blocking peptides to mimic the 110–135 domain of CIRP. Each peptide was modified by appending an additional Tat transmembrane functional domain (YGRKKRRQRRR), resulting in the formation of Tat‐C16‐1 (YGRKKRRQRRR‐RGRGFSRGGGDRGYGG), Tat‐C16‐2 (YGRKKRRQRRR‐SRGGGDRGYGGGRFES), and Tat‐C16‐3 (YGRKKRRQRRR‐DRGYGGGRFESRSGGY). These Tat‐fused peptides (Tat‐C16‐1, Tat‐C16‐2, and Tat‐C16‐3) were custom synthesized by Hefei KS‐V Peptide Biotechnology (Hefei, China). We subsequently performed in vitro validation of these interactions and observed that Tat‐C16‐1 exhibited the most pronounced interaction with GluR1. Consequently, we utilized Tat‐C16‐1 peptide for our subsequent studies and, for simplicity, referred to it as Tat‐C16 in all subsequent analyses. The Tat‐C16 peptide was administered intravenously at a dosage of 20 mg/kg following 7 days of HH exposure.

### 2.2 Cell culture

Primary hippocampal neurons were harvested from E18 C57BL/6 mice embryos. The lower abdomen of the embryos was sterilized with 70% ethanol before quickly transferring them into HBSS (China) for dissection. The hippocampal tissues were carefully isolated, with meticulous removal of meninges and blood vessels, followed by trypsin digestion. The neuronal cells were then cultured in Neurobasal medium enriched with B27 (Gibco), Glutamax (Gibco), and PS (Hyclone), with media changes every 3 days. These primary hippocampal neurons were exposed to 1% O_2_ to simulate hypoxic conditions.

### 2.3 Neurobehavioral test

#### 2.3.1 Morris water maze (MWM) test

The MWM test was prepared by filling a pool with water maintained at an environmental temperature of 22–24°C and adding white pigment to enhance visibility. The water was stirred until uniformity was achieved, with the platform positioned 1 cm above the water surface on the first day and submerged for the remaining training days. During the final test phase, the platform was removed. Mice were introduced into the pool facing the wall, and their ability to locate the visible or submerged platform within 60 s was recorded. Mice unable to find the platform within the allotted time were guided to it and allowed to remain for 20 s. Performance metrics were captured using ANY‐maze video tracking software (Stoelting Co), which was also used to obtain statistical data. Latency was defined as the time when the mouse first crossed the platform position, while entries represented the number of times a mouse crossed a platform location. The swimming distance and swimming time of mice in the target quadrant were calculated as the proportion of the total swimming distance (% distance traveled in the target quadrant) and total swimming time (Target quadrant occupancy), respectively.

#### 2.3.2 Training schedule and methods

Day 1–Day 5: Training with the platform located in the southwest (SW) quadrant, starting from different locations (NW, NE, SW, and NW) for each trial. Day 6: No platform present, to assess memory retention.

#### 2.3.3 Step‐down inhibitory avoidance test (SIAT)

For the SIAT, we utilized the SA202 rat and mouse step‐down system (Saiangsi). Initially, animals were acclimated to the platform, undergoing three 10 s acclimation sessions with 30 min intervals between each. Following acclimation, training commenced immediately. Mice received a 0.5 mA plantar electric shock with a 2 s delay upon leaving the platform. The initial test was conducted at the end of the training session, with a follow‐up test 24 h later to assess memory retention.

#### 2.3.4 Step‐through active avoidance test (SAAT)

The SAAT was performed using the SA222 dark avoidance shuttle box (Saiangsi). Mice were placed in the apparatus and allowed to adapt. Subsequently, an acousto‐optic stimulus (a combination of high‐frequency sound and flashing light) was presented, followed by a 0.5 mA plantar electroshock after 5 s. Training continued until mice achieved an 80% success rate in avoiding the shock. Tests conducted 24 and 48 h post‐training evaluated the mice's ability to avoid the shock, recording both successful avoidances and errors.

### 2.4 Golgi staining

For Golgi staining, mice were anesthetized, and brains were extracted without perfusion. Brain samples were processed using the HitoBiotec Golgi staining kit (HTKNS1125NH, Hitobiotec Corp) and sectioned at 100 μm thickness using a vibrating slicer (VT1000 S, Leica). Sections were air‐dried overnight at room temperature in a dark, cool environment. The staining process involved two initial rinses in distilled water for 3 min each, followed by a 10 min stain with the kit's solution. After staining, sections were rinsed again twice in distilled water for 4 min each, dehydrated through a graded ethanol series (50%, 70%, and 95% for 5 min each), and cleared in xylene for 5 min. After three rounds of dehydration in anhydrous ethanol for 5 min each and final xylene treatment, sections were mounted with neutral gum. Imaging was performed once the xylene had evaporated and the sections had dried. Lastly, the statistical analysis of dendritic numbers relied on the Neuron J plug‐in (accessible via Plugins→Neuron J) of Image J (NIH, USA), while the statistical method for counting spine numbers involved selecting 10 μm neuron branches using the Cell Counter tool (accessible via Plugins→Analyze→Cell Counter).

### 2.5 Real‐time quantitative PCR (qPCR)

RNA extraction was performed using Trizol (9109, Takara), and cDNA synthesis was achieved with a Real‐time PCR kit (R323‐01, Vazyme). Quantitative PCR was conducted using AceQ qPCR SYBR Green Master Mix (Q141‐02, Vazyme) according to the manufacturer's instructions on a StepOnePlus PCR system (Thermo). Specific primers were used to amplify Mus musculus *Gria1*, *Gria2*, and *Cirp* (*Cirbp*), with sequences as follows: *Gria1* primers 5′‐CCTACATCGTCACGACTATCCTC‐3′ and 5′‐AGTTCCACGCAGTAGCCCTCAT‐3′; *Gria2* primers 5′‐TGCATAGTCCTTAGGATCCCCT‐3′ and 5′‐TGCTGCTCCTACACAACCTT‐3′; *Cirbp* primers 5′‐GCAGGTCTTCTCCAAGTATGGG‐3′ and 5′‐ATGGCGTCCTTAGCGTCATCGA‐3′. Mus musculus *β‐actin*, amplified with primers 5′‐AAATCGTGCGTGACATCAAAGA‐3′ and 5′‐GCCATCTCCTGCTCGAAGTC‐3′, served as the loading control.

### 2.6 Tissue immunofluorescence staining

Mice were deeply anesthetized and perfused with 0.9% saline before brain extraction and fixation in 4% PFA (MERYER) for over 24 h. Post‐fixation, tissues were dehydrated in 30% sucrose solution. Hippocampal sections (40 μm thick) were prepared, washed with PBS, permeabilized with 0.3% TritonX‐100, and blocked with 10% BSA. Overnight incubation at 4°C with primary antibodies against PSD95 (CST3450, USA), GluR1 (AbcamAb109450, China), CIRP (Proteintech10209‐2‐AP, USA), NeuN (MilliporeMAB377, USA), GFAP (CST3670, USA), and Iba1 (FUJIFILM016‐20001, Japan) was followed by secondary antibody incubation. DAPI (1 μg/μL, Abmole, USA) was used for nuclear staining. Images were captured using an SP8 confocal microscope (Leica). Subsequently, these images were converted to 8‐bit format using Image J for statistical analysis. The process involved several steps: first, adjusting the threshold by navigating to Image→Adjust→Threshold and setting the default parameters. Second, selecting the parameters of interest, including Area, Integrated density, Standard deviation, Mean gray value, and enabling the “Limit to threshold” option by going to Analyze→Set measurements. Third, measuring the relevant parameters (such as the area, integrated density, etc.) using Analyze→Measure. Finally, the AU was calculated using the formula Mean = integrated density/area, and the results were normalized.

### 2.7 Plasmid transfection and luciferase assay

GluR1 plasmids (pm‐GLO‐GluR1‐1 and pm‐GLO‐GluR1‐2) were designed based on potential CIRP binding sites identified via the catRAPID platform. HEK293T cells in a 24‐well plate were transfected with 800 ng of plasmid, 50 μL Opti‐MEM™ (Gibco), and 2 μL of Lipofectamine™ 2000 (Invitrogen™) for 48 h. Luciferase activity was measured using Promega's Luciferase Assay System (E1500). Cells were lysed and transferred to EP tubes, with 25 μL of cell lysis solution and 25 μL of reaction reagent added to each assay well. Absorbance was read at 540 nm before and after adding 25 μL of the reaction termination solution.

### 2.8 Protein and synaptosomes extraction

Total protein from mouse brain tissue was isolated using a lysis buffer containing protease inhibitors, with cytoplasmic and membrane proteins further separated using a kit from Beyotime Biotechnology. Synaptosomes were prepared by homogenizing brain tissue in a buffer (pH 7.4, containing 320 mM sucrose, 5 mM sodium pyrophosphate, 1 mM EDTA, 10 mM Hepes, 200 nM okadaic acid, and a protease inhibitor mix), followed by differential centrifugation. The final pellet, obtained after ultracentrifugation through a nonlinear sucrose gradient, was resuspended in a lysis solution with protease and phosphatase inhibitors. Protein concentrations were quantified using the BCA kit (Thermo).

### 2.9 Co‐immunoprecipitation (Co‐IP)

Proteins were lysed in non‐denaturing buffer from the Beyotime immunoprecipitation kit (P2179S, China), and concentrations were standardized across samples. Proteins were incubated with Protein A/G Beads, followed by overnight incubation with IP antibodies (GluR1, AbcamAb109450, USA; CIRP, Proteintech10209‐2‐AP, USA; or IgG, Jackson12479, USA) at 4°C. After heating with loading buffer and magnetic separation, the supernatant was collected for Western blot analysis.

### 2.10 Western blot

Proteins were separated by SDS‐PAGE and transferred to PVDF membranes (ROCHE, CH). Membranes were blocked with TBST containing 5% skim milk and incubated with primary antibodies against GluR1, PSD95, CIRP, NaKATPase, HSP90β, and Tubulin overnight at 4°C. Following primary antibody incubation, membranes were washed and incubated with a secondary antibody at 1:10000 dilution. Chemiluminescent detection was performed (Tanon 4100, China), and band intensities were analyzed using Image J.

### 2.11 Data processing and statistics

All data were subjected to tests for normality. Behavioral data were presented as mean ± SEM, while other experimental data were shown as mean ± SD. Statistical analyses were conducted using unpaired *t*‐tests for two‐group comparisons and one/two‐way ANOVA with Bonferroni post‐tests for multiple group comparisons. A *p*‐value <0.05 was considered statistically significant. Analyses were performed using GraphPad Prism 8.3 (GraphPad Software).

## 3 RESULTS

### 3.1 Impact of HH on cognitive function and synaptic structure

**FIGURE 1:**
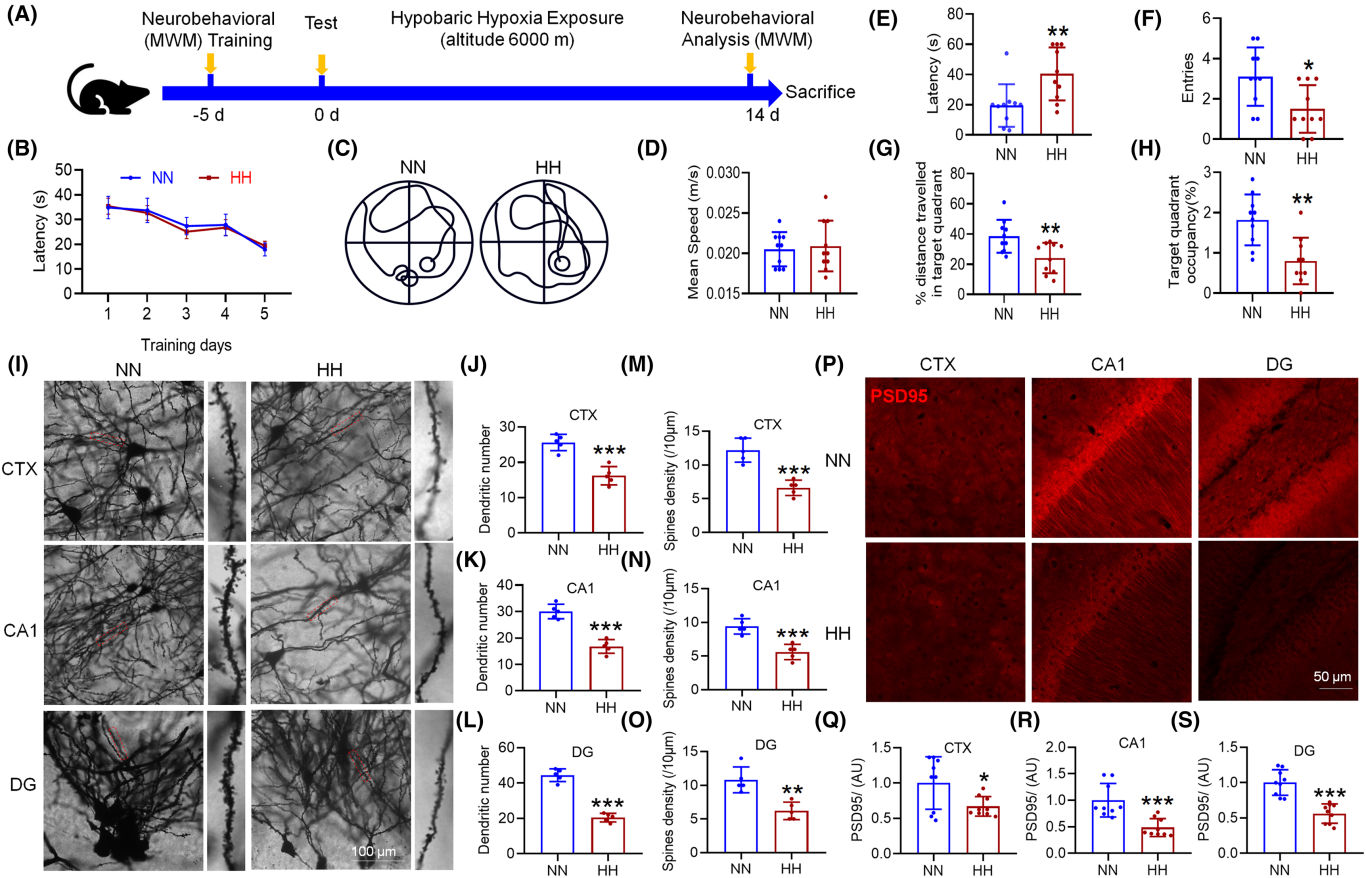
Impact of HH on learning, memory, and synaptic integrity in mice. (A) Morris water maze setup illustrating the experimental design for assessing spatial learning and memory. (B) Time to locate the hidden platform during the initial training phase over 5 days. (C). (D) Average swimming speed, indicating motor function during the evaluation phase. (E) Latency to first locate the hidden platform during the evaluation phase, a measure of memory recall. (F) Frequency of crossing the former platform location, reflecting memory accuracy. (G) Percentage of total swim distance within the target quadrant, assessing spatial memory retention. (H) Percentage of total swim time within the target quadrant, further evaluating spatial memory. (I) Golgi staining reveals dendritic spine morphology post‐14 days HH exposure, with subsequent quantitative analysis of dendritic branches and spine density in the cortex (CTX) (J,M), CA1 (K,N), and DG (L,O) regions. (P) Immunofluorescence detection of PSD95 protein distribution in the brain post‐HH exposure, with fluorescence intensity quantification in the CTX (Q), CA1 (R), and DG (S) regions. Significance indicated as *p < 0.05, **p < 0.01, ***p < 0.001 compared to normobaric normoxia (NN) control, *n* = 5 ~ 10.

We investigated the effects of HH on cognitive function and synaptic morphology in mice using the MWM to assess spatial memory capabilities (Figure 1A–C). Our findings revealed no significant difference in swimming speed among the groups (Figure 1D), indicating that motor skills were not affected by HH exposure. However, mice subjected to HH displayed a notable decline in their ability to locate hidden platforms, evidenced by increased escape latencies (Figure 1E), and a reduction in the number of times they crossed the original platform location, as well as total swimming time and distance in the target quadrant (Figure 1F–H). These results suggest that HH exposure adversely affects memory function in mice. Further analysis focused on dendritic spine morphology, a key component of synaptic plasticity and cognitive function. Post‐HH exposure, there was a significant reduction in the number of neuronal branches and dendritic spines in the brain tissue (Figure 1I–O), alongside a marked decrease in PSD95 protein expression within the cortex, hippocampus CA1 and dentate gyrus (DG) regions of the hippocampus (Figure 1P–S). These changes indicate a detrimental effect of HH on synaptic integrity, highlighting the critical role of synaptic structure in learning and memory processes.

### 3.2 HH modulates the GluR1 and CIRP expression and localization

**FIGURE 2:**
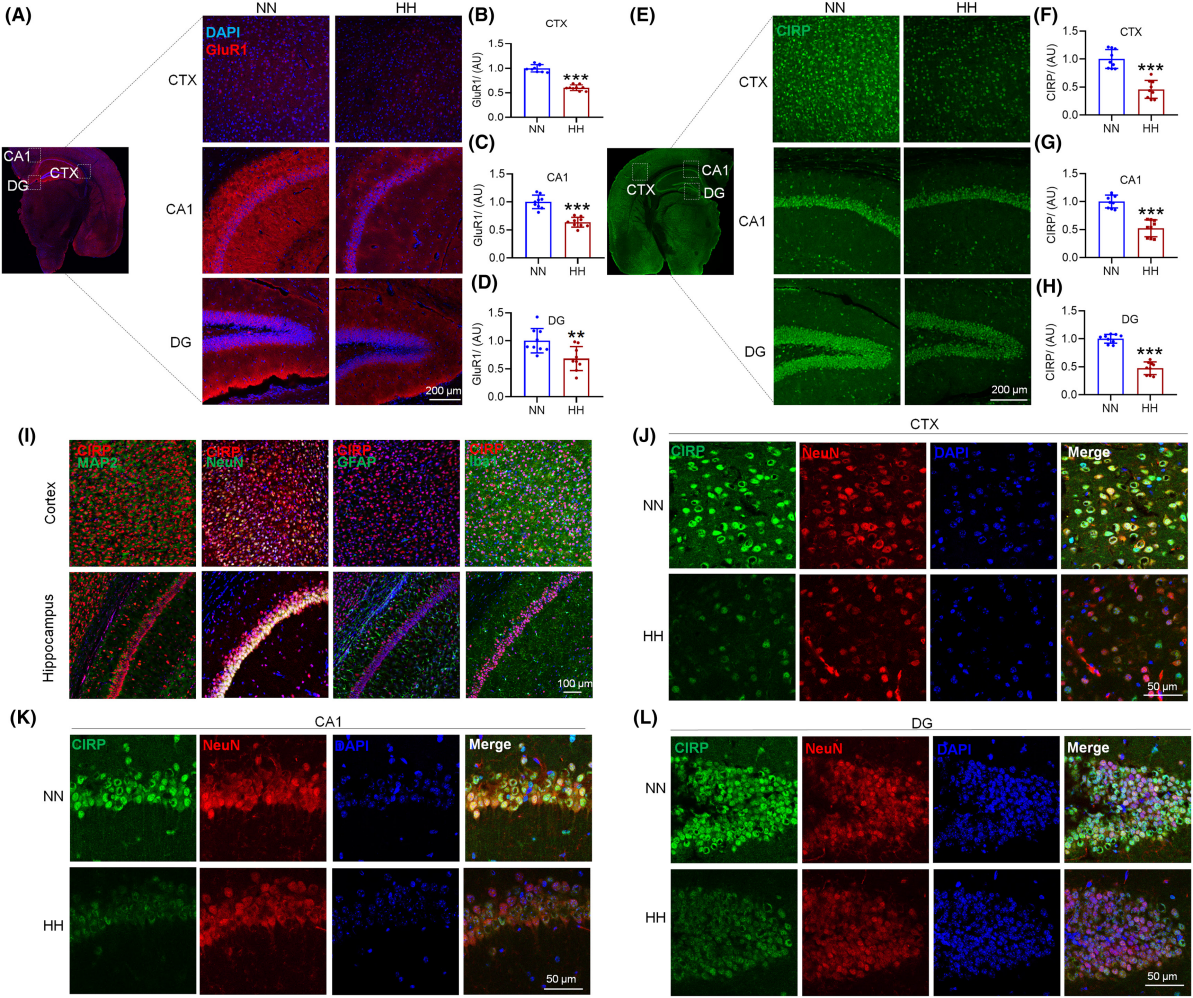
HH alters GluR1 and CIRP levels in the mouse brain. (A) Immunofluorescence reveals changes in GluR1 expression and localization within the CTX, CA1, and DG after 14 days of HH exposure. Quantitative analysis of GluR1 fluorescence intensity is shown for the CTX (B), CA1 (C), and DG (D). (E) Immunofluorescence also highlights CIRP expression and distribution changes in the CTX, CA1, and DG regions following HH exposure, with corresponding fluorescence intensity quantifications in the CTX (F), CA1 (G), and DG (H). (I) Co‐localization studies of CIRP with neuronal marker MAP2, astrocytic marker GFAP, and microglial marker Iba1 demonstrate CIRP's diverse cellular interactions in the cortex and hippocampus. Detailed views of CIRP expression and localization in neuronal cells of the CTX (J), CA1 (K), and DG (L) regions post‐HH exposure are also provided. Significance levels are indicated as *p < 0.05, **p < 0.01, ***p < 0.001 compared to NN control, *n* = 3.

Investigating the impact of 14 days of HH exposure, we analyzed the alterations in GluR1 and CIRP within neuronal dendritic spines. Immunofluorescence studies highlighted changes in GluR1 (Figure 2A) and CIRP (Figure 2E) across the cortex and hippocampus CA1 and DG. Immunofluorescence quantitative assessments revealed a significant reduction in both GluR1 (Figure 2B–D) and CIRP (Figure 2F–H) protein levels in these brain regions under HH. The co‐localization of CIRP with neuronal marker MAP2, astrocyte marker GFAP, and microglia marker Iba1 in the cortex and hippocampus indicates that the cellular distribution of CIRP is mainly in neurons (Figure 2I). After exposure to HH, all the expression of CIRP in neuronal cells in the cortex (Figure 2J) and hippocampal CA1 (Figure 2K) and DG (Figure 2L) regions decreased. CIRP localization was predominantly observed in the neuronal nucleus, with an absence in astrocytes and microglia, indicating a neuron‐specific response to HH.

**FIGURE 3:**
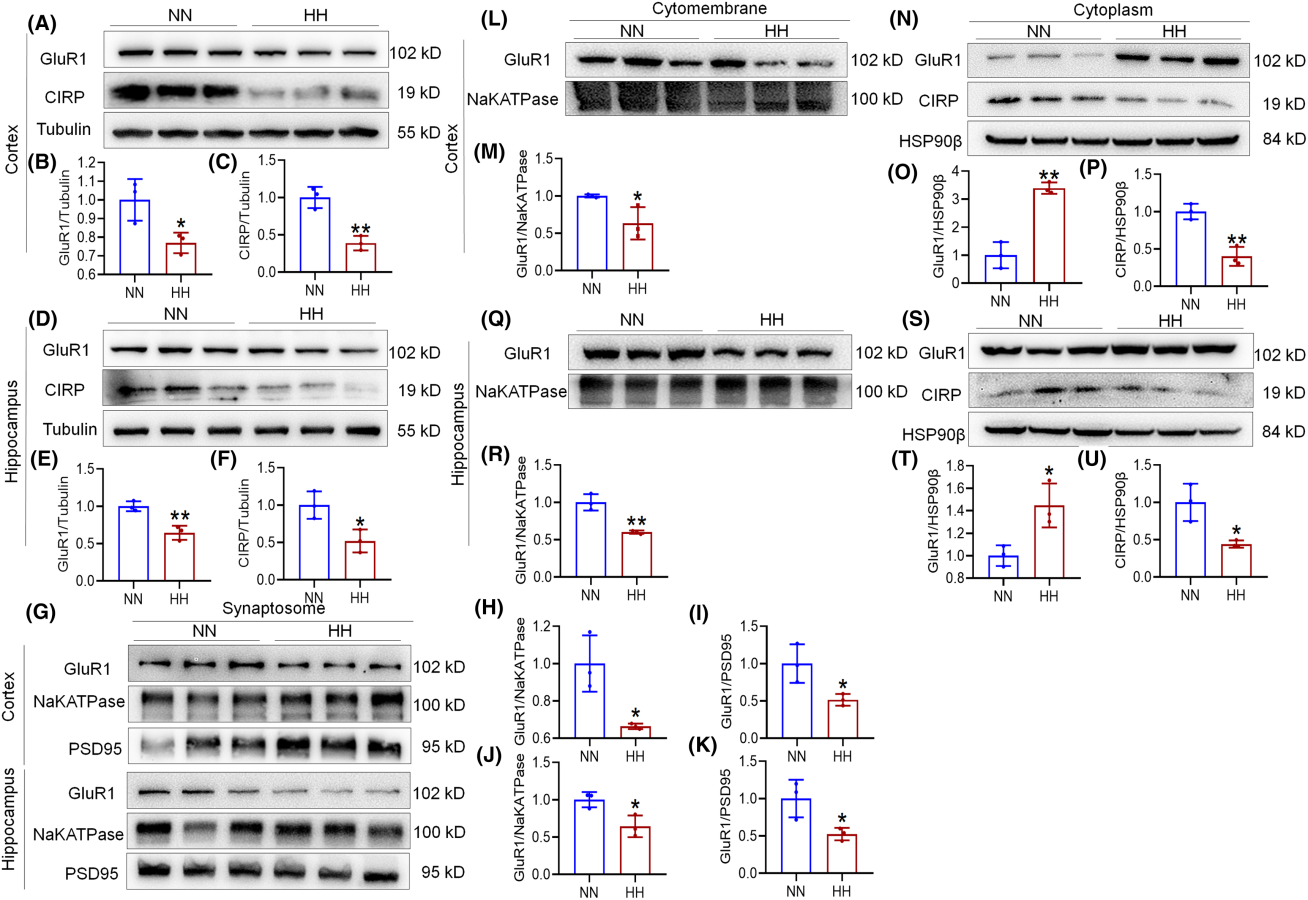
HH modulates GluR1 and CIRP protein levels in mouse brain. (A,D) Western blot analyses reveal the expression patterns of GluR1 and CIRP in the cortex and hippocampus following 14 days of HH exposure. (B,E,C,F) Quantitative evaluations depict the expression levels of GluR1 and CIRP in these brain regions, respectively. (G) Investigation of GluR1 alterations in synaptosomes from the cortex and hippocampus under HH conditions. (H–K) Quantitative analysis of GluR1 levels in synaptosomal preparations. (L,Q) Examination of GluR1 in the membrane fractions of the cortex and hippocampus post‐HH exposure. (N,S) Analysis of GluR1 and CIRP in the cytosolic fractions of these brain regions following HH. (M,R,O,T,P,U) Statistical representations of GluR1 and CIRP levels in the membrane and cytosolic fractions, highlighting the impact of HH on protein distribution and expression. Significance is marked as *p < 0.05, **p < 0.01, ***p < 0.001 when compared to the NN control group, *n* = 9.

Consistent with the immunofluorescence findings, the Western blot analysis revealed a reduction in GluR1 and CIRP protein levels in both the cortex (Figure 3A–C) and hippocampus (Figure 3D–F). Contrarily, mRNA analysis showed no significant change in *Gria1* and *Gria2* expressions (Figure S1A,B,D,E), while *Crip* mRNA exhibited a notable increase (Figure S1C,F), suggesting a potential compensatory mechanism. Further analyses showed a decrease in GluR1 expression within synaptosomes, indicating disrupted synaptic function (Figure 3G–K). GluR1 was found to relocate from the neuronal membrane to the cytoplasm following HH exposure, underscoring synaptic disruption (Figure 3L–U). Additionally, primary neuron studies after 1% O_2_ hypoxia treatment revealed a similar pattern of GluR1 translocation from the membrane to the cytoplasm over various durations (0 h, 3 h, 6 h, and 12 h) (Figure S1G,H), aligning with the observed decrease in *Gria1* mRNA levels alongside *Crip* (Figure S1I,J). These findings collectively demonstrate that HH exposure significantly impacts the expression and distribution of GluR1 and CIRP, affecting synaptic integrity and potentially altering neuronal function.

### 3.3 CIRP modulates GluR1 protein interaction and expression

**FIGURE 4:**
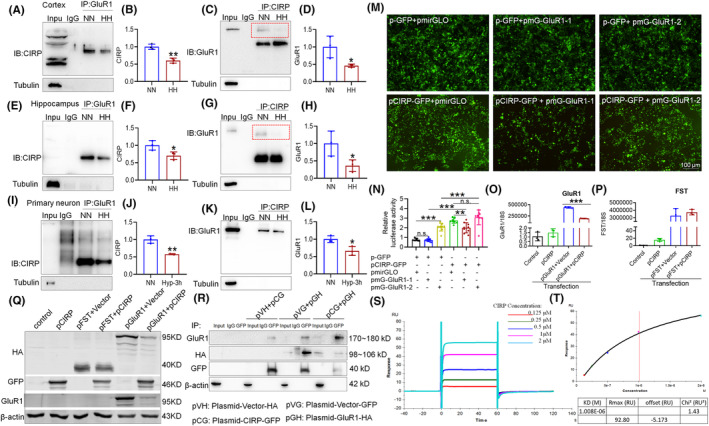
Reduced CIRP‐GluR1 protein interaction following HH in mice. (A,C,E,G) Co‐immunoprecipitation (Co‐IP) analysis reveals diminished interactions between GluR1 and CIRP proteins in the cortex and hippocampus after 14 days of HH exposure and 3 h of 1% O_2_ hypoxic conditions (I,K). (B,D,F,H,J,L) Quantitative assessments show the interaction levels between GluR1 and CIRP in these brain regions under both 14 days HH and short‐term hypoxic exposure. (M) Visualization of GFP‐tagged protein expression in HEK293T cells transfected with various plasmids for 48 h. (N) Luciferase activity measured in cells co‐transfected with pCIRP‐GFP and pmG‐GluR1‐1 or pmG‐GluR1‐2, indicating the effect of CIRP on GluR1 expression. (O‐P) qPCR analysis details mRNA expression changes in cells overexpressing CIRP and GluR1. (Q) Western blot (WB) analysis evaluates the protein expression related to this interaction. (R) Further Co‐IP analysis confirms the binding between CIRP and GluR1 proteins. (S,T) Surface plasmon resonance (SPR) provides detailed binding analysis and affinity calculations for CIRP's interaction with the GluR1 carboxyl‐terminal sequence. Significance denoted as *p < 0.05, **p < 0.01, ***p < 0.001 compared to NN control, *n* = 9.

Our study reveals that CIRP plays a dual role in modulating the AMPA receptor subunit GluR1, both by interacting with it and by influencing its expression levels. Co‐IP analyses demonstrated a significant reduction in the interaction between CIRP and GluR1 within the cortex and hippocampus of mouse brain tissue after 14 days of HH exposure (Figure 4A–H), a finding corroborated in neuronal cells subjected to 1% O_2_ for 3 h (Figure 4I–L). Further investigation into the potential binding sites between CIRP and GluR1, guided by catRAPID predictions (Figure S2A,B), led to the identification of two fragments with high binding affinity. These fragments were used to construct plasmids for transfection into HEK293T cells. Luciferase assay results indicated that a plasmid overexpressing CIRP significantly reduced the expression of one GluR1 construct (pmG‐GluR1‐1) (Figure S2C,D), suggesting a specific inhibitory effect of CIRP on the GluR1 expression. To control for potential artifacts related to the fusion protein nature of the CIRP overexpression plasmid, additional analyses were performed. These showed that while a GFP tag alone could enhance the luciferase expression of PMG‐GluR1‐2, the CIRP overexpression plasmid specifically inhibited PMG‐GluR1‐1 expression (Figure 4M,N). Subsequent qPCR and Western blot assays confirmed that elevated CIRP levels significantly reduced GluR1 mRNA and protein expression (Figure 4O,Q), without affecting *FST* mRNA (Figure 4P), serving as a negative control. In vitro Co‐IP further validated the direct interaction between CIRP and GluR1 (Figure 4R), and surface plasmon resonance (SPR) analysis detected moderate binding affinity between full‐length CIRP protein and the GluR1 carboxy‐terminal region (Figure 4S,T). These findings underscore the intricate regulatory role of CIRP in GluR1‐mediated synaptic plasticity, particularly under conditions of hypoxic stress.

### 3.4 *Cirp* knockout mice display cognitive impairments and altered synaptic morphology

**FIGURE 5:**
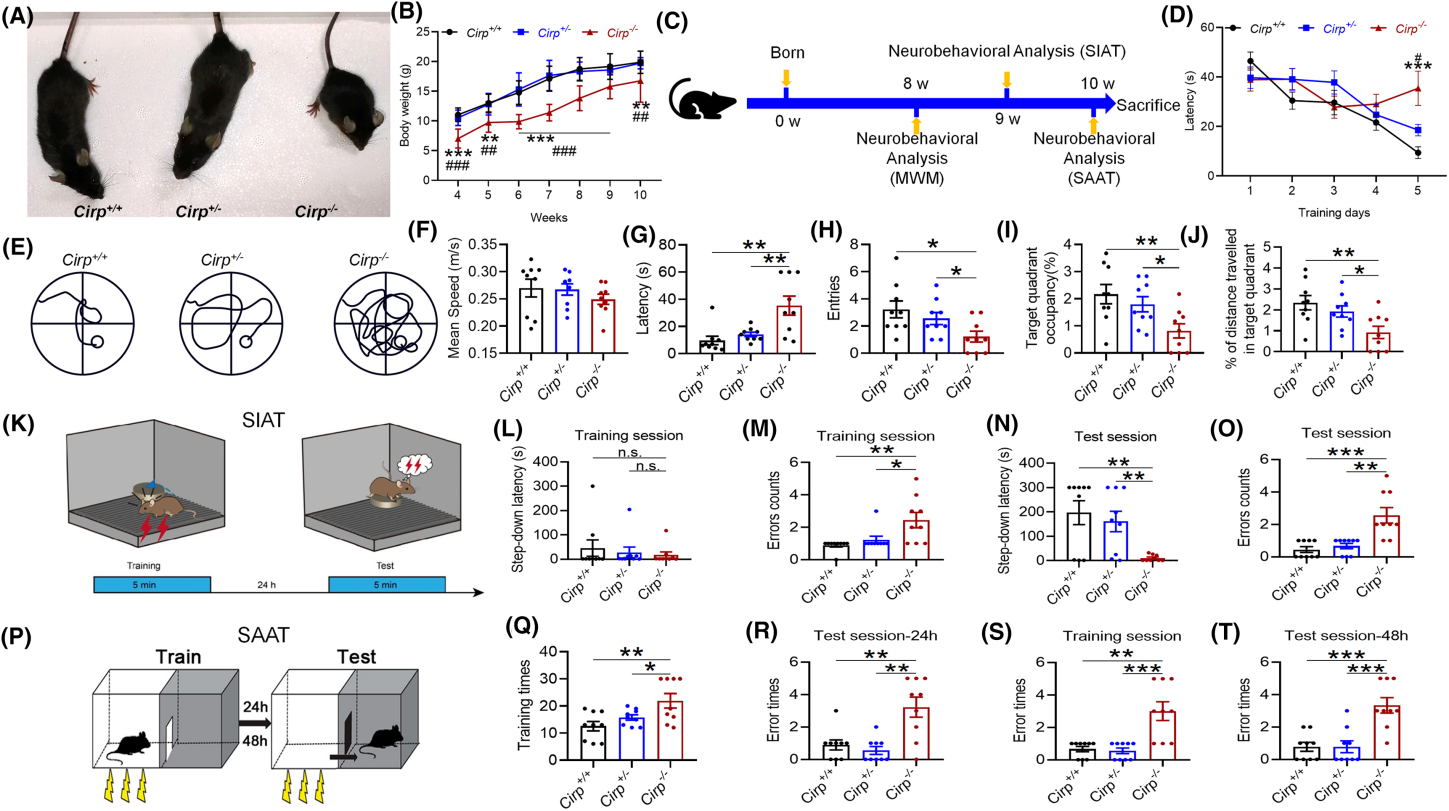
Comparative analysis of phenotype, body weight, and cognitive performance across *Cirp* genotypes. (A) Visual representation of mice across different *Cirp* genotypes. (B) Body weight progression from 4 to 10 weeks, highlighting differences between *Cirp* ^+/+^ and *Cirp* ^−/−^ groups (*), and *Cirp* ^+/−^ and *Cirp* ^−/−^ groups (#). (C) Experimental timeline: *Cirp* ^+/−^ mice underwent Morris water maze testing at 8 weeks, step‐down testing at 9 weeks, and active avoidance testing at 10 weeks. (D) Morris water maze test phase swim paths. (E) Latency to locate the platform during initial training. (F) Average swim speed during the test phase. (G) Latency to platform discovery during the test phase. (H) Platform location crossings during the test phase. (I) Time spent in the target quadrant during the test phase. (J) Distance covered in the target quadrant during the test phase. (K) Step‐down test setup schematic. (L,N) Latency to step down during the step‐down test's training and test phases. (M,O) Errors committed during the step‐down test's training and test phases. (P) Active avoidance test setup schematic. (Q) Training trials required to reach an 80% success rate in the active avoidance test. (R–T) Errors during the active avoidance test's training and 24 h, 48 h test phases. Significance levels: *p < 0.05, **p < 0.01, ***p < 0.001 compared to the indicated group; ^##^ *p* < 0.01, ^###^ *p* < 0.001, n = 9.

**FIGURE 6:**
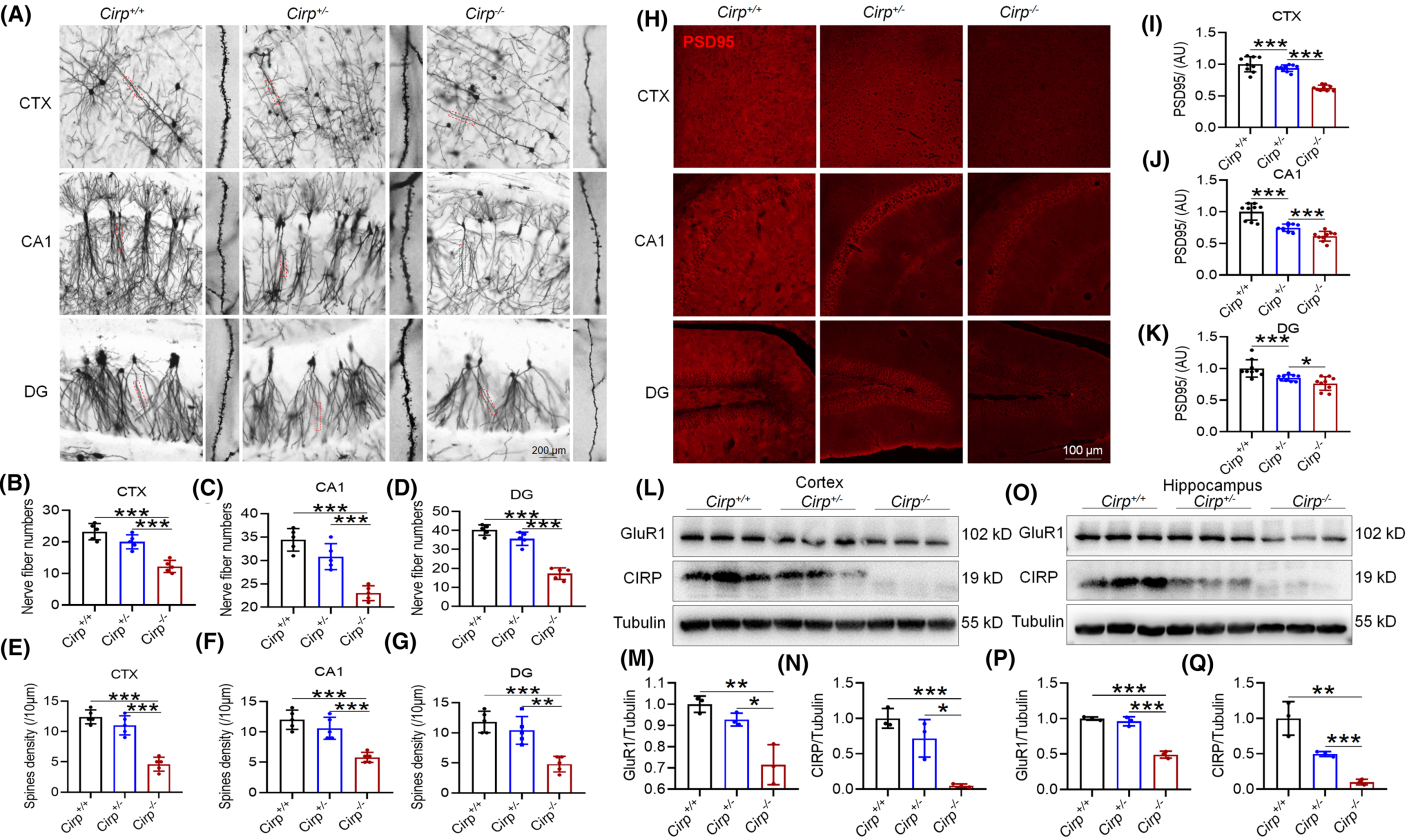
Synaptic architecture and protein expression across *Cirp* genotypes in mice. (A) Golgi staining showcases dendritic spine morphology in neurons from mice of varying *Cirp* genotypes. (B–D) Quantification of neuronal branch numbers across genotypes. (E–G) Analysis of dendritic spine density, highlighting differences among *Cirp* genotypes. (H) Immunofluorescence reveals PSD95 protein expression and localization in the CTX, CA1, and DG regions. (I–K) Quantitative assessment of PSD95 fluorescence intensity in CTX, CA1, and DG, illustrating synaptic protein distribution. (L,O) Western blot analysis evaluates GluR1 and CIRP protein levels in the cortex and hippocampus. (M,N, P,Q) Statistical representation of GluR1 and CIRP expression, comparing across brain regions and genotypes. Significance denoted as *p < 0.05, **p < 0.01, ***p < 0.001 relative to the corresponding control group, *n* = 5.

In our study involving *Cirp* knockout (KO) mice, we observed significant developmental and cognitive differences when compared to their *Cirp* ^+/+^ and *Cirp* ^+/−^ counterparts. Post‐birth, *Cirp* ^−/−^ mice demonstrated slower growth rates, smaller body sizes, and lower body weights (Figure 5A,B). Behavioral assessments, including the MWM, revealed that *Cirp* ^−/−^ mice performed similarly to mice subjected to 14 days of HH, indicating impaired spatial learning and memory capabilities (Figure 5D–J). Further cognitive testing through the SIAT and the SAAT showed that *Cirp* ^−/−^ mice exhibited significantly poorer memory retention, as evidenced by a higher number of errors compared to the control groups (Figure 5K–T). Morphological analysis of neuronal dendritic spines in 8‐week‐old mice, conducted via Golgi staining and immunofluorescence, revealed a marked reduction in both the number of neuronal branches and the density of dendritic spines in the brains of *Cirp* ^−/−^ mice (Figure 6A–G). Additionally, the expression of PSD95, a protein critical for synaptic stability, was significantly lower in the cortex, CA1, and DG regions of the hippocampus in *Cirp* ^−/−^ mice (Figure 6H–K). This reduction in synaptic components was paralleled by decreased expression levels of GluR1 and CIRP proteins in both the cortex and hippocampus (Figure 6L–Q). These findings suggest that the absence of *Cirp* not only affects the physical development of mice but also significantly impairs their cognitive functions. The observed reductions in dendritic spine density and synaptic protein expression highlight the crucial role of CIRP in maintaining synaptic integrity and, by extension, learning and memory processes.

### 3.5 Decreased GluR1 levels and altered distribution in *Cirp* knockout mice

**FIGURE 7:**
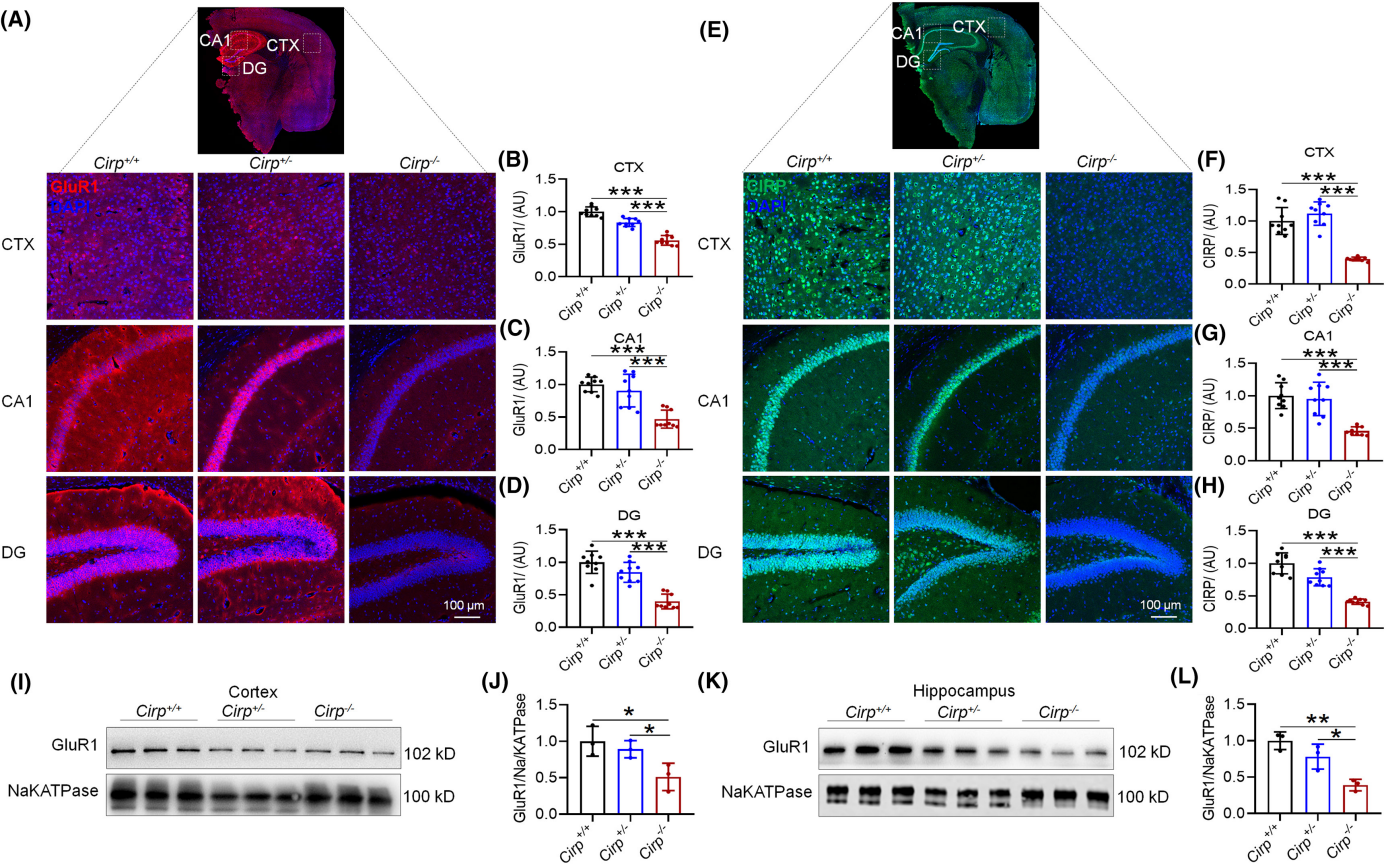
Comparative analysis of GluR1 and CIRP expression in mice across *Cirp* genotypes. (A) Immunofluorescence (IF) showcases GluR1 protein localization in the cortex and hippocampus, illustrating genotype‐dependent expression patterns. (B–D) Quantitative analysis of GluR1 fluorescence intensity reveals significant variations in the cortex, CA1 region, and overall hippocampus among different genotypes. (E) IF imaging of CIRP protein distribution in the cortex and hippocampus highlights differences in expression across genotypes. (F–H) Statistical evaluations of CIRP fluorescence intensity in the cortex, CA1 region, and hippocampus, indicating genotype‐specific expression levels. (I,K) Western blot analysis of GluR1 in synaptosomes from the cortex and hippocampus underscores the impact of *Cirp* genotype on GluR1 protein levels. (J,L) Quantitative assessments of GluR1 expression in the cortex and hippocampus via Western blot, demonstrating significant differences among genotypes. Significance is denoted as *p < 0.05, **p < 0.01 relative to the corresponding control group, *n* = 9.

**FIGURE 8:**
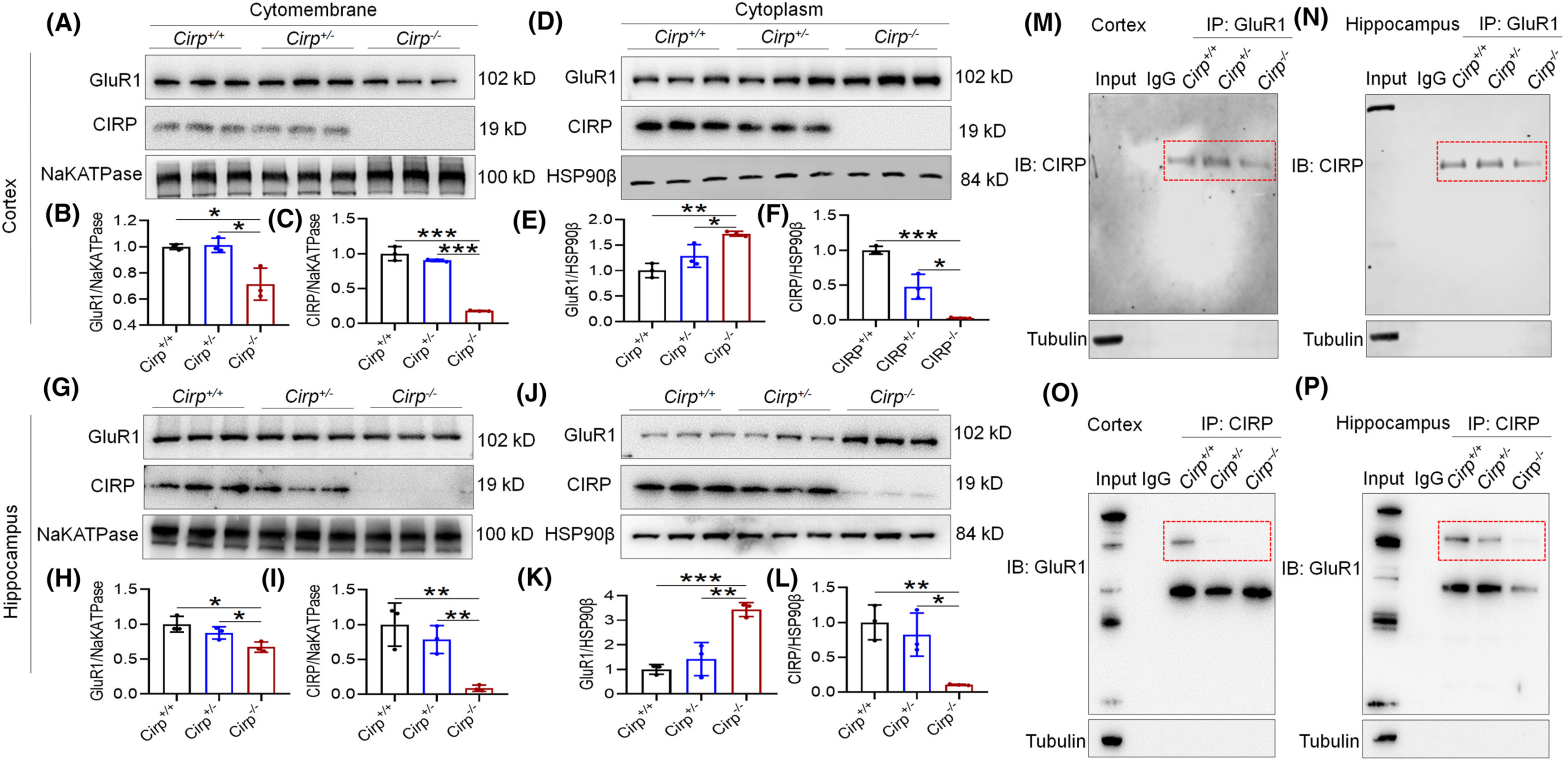
Differential expression and interaction of GluR1 and CIRP across *Cirp* genotypes in mouse brain. (A,G) Western blot analyses reveal variations in GluR1 and CIRP protein levels on the cell membranes within the cortex and hippocampus. (D,J) Examination of changes in GluR1 and CIRP protein levels within the cytoplasm of these brain regions. (B,C, E,F) Quantitative assessments of GluR1 and CIRP membrane expression in the cortex and hippocampus, as determined by Western blot. (H,I, K,L) Statistical evaluations of cytoplasmic GluR1 and CIRP levels in the cortex and hippocampus. Co‐immunoprecipitation (Co‐IP) analyses demonstrate the interactions between GluR1 and CIRP in the cortex (M,O) and hippocampus (N,P), with IP targeting GluR1 and CIRP respectively, and IB detecting the reciprocal protein. Significance levels are indicated as *p < 0.05, **p < 0.01, ***p < 0.001 relative to the control group, *n* = 9.

Our analysis through Western blot and immunofluorescence revealed a significant reduction in GluR1 protein levels in the brain tissue of *Cirp* ^−/−^ mice, compared to controls (Figure 7A–D). Additionally, CIRP protein levels were markedly lower in *Cirp* ^−/−^ mice than in *Cirp* ^+/+^ and *Cirp* ^+/−^ groups (Figure 7E–H). This reduction extended to the brain's synaptosomes, where GluR1 expression was also significantly diminished in *Cirp* ^−/−^ mice (Figure 7I–L). Further investigation into the distribution of GluR1 within the brain tissue of *Cirp* ^−/−^ mice revealed a pattern similar to that observed in mice subjected to 14 days of HH, with both GluR1 and CIRP protein expression levels decreased (Figure 8A–L). Co‐IP analysis confirmed a significant reduction in the interaction between GluR1 and CIRP proteins in the brain tissue of *Cirp* ^−/−^ mice (Figure 8M–P). These findings suggest that the absence of *Cirp* not only leads to reduced GluR1 expression but also affects its distribution within the brain, mirroring changes seen under hypoxic conditions. This reduction in GluR1 and its interaction with CIRP could contribute to the cognitive deficits observed in *Cirp* ^−/−^ mice, highlighting the critical role of these proteins in maintaining normal brain function and synaptic integrity.

### 3.6 Tat‐C16 peptide mitigates cognitive decline induced by HH

Our investigation into the therapeutic potential of the Tat‐C16 peptide against HH‐induced cognitive impairments began with identifying the binding sites between CIRP and the GluR1 protein. Analysis via the PDBe website revealed that CIRP predominantly binds to the GluR1 sequence between amino acids 563–894, encompassing key dephosphorylation sites at positions 831 and 845 (Figure S3A–D). We subsequently employed a truncation strategy, dividing the target segments into 16‐amino‐acid sections and attaching a Tat tag to the N‐terminus of each truncated sequence. This approach led to the synthesis of three peptides: Tat‐C16‐1 (spanning amino acids 110–125), Tat‐C16‐2 (115–130), and Tat‐C16‐3 (120–135). Western Blot analysis of membrane proteins revealed that Tat‐C16‐1 exhibited superior performance, notably enhancing GluR1 stability on neuronal membranes under hypoxic conditions (Figure S3E). Consequently, Tat‐C16‐1 was chosen for further experimentation.

**FIGURE 9:**
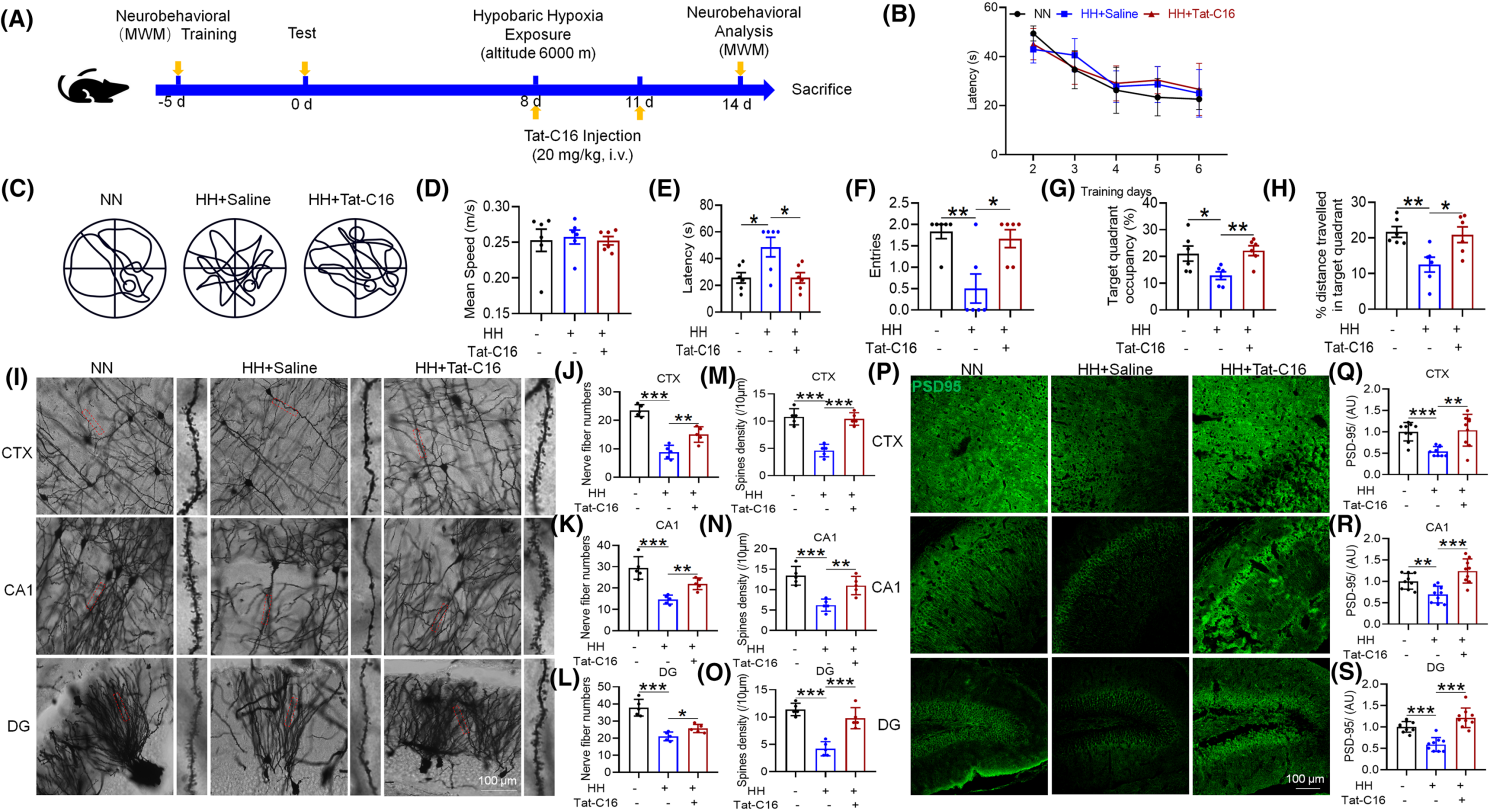
Impact of Tat‐C16 peptide on learning, memory, and synaptic integrity under HH. (A) Experimental timeline detailing pre‐treatment MWM training, Tat‐C16 peptide administration on days 8 and 11 of HH exposure, and post‐treatment MWM evaluation. (B) Latency to initially locate the platform during the training phase. (C) Swim paths during the test phase, illustrating navigational strategies. (D) Mean swim speed, indicating motor function during the test phase. (E) Latency to platform discovery in the test phase, assessing memory recall. (F) Frequency of original platform location crossings during the test phase, reflecting memory precision. (G) Time percentage spent in the target quadrant during the test phase, evaluating spatial memory retention. (H) Distance percentage covered in the target quadrant, further assessing spatial memory. (I) Golgi staining visualizes dendritic spine morphology after 14 days of HH exposure. (J–L) Quantitative analysis of neuronal branch numbers. (M–O) Dendritic spine density statistics. (P) Immunofluorescence highlights PSD95 protein distribution in the cortex and hippocampus post‐HH exposure. (Q–S) Quantification of PSD95 fluorescence intensity in the CTX, CA1, and DG regions. Significance denoted as *p < 0.05, **p < 0.01, ***p < 0.001 relative to the control group, *n* = 6.

**FIGURE 10:**
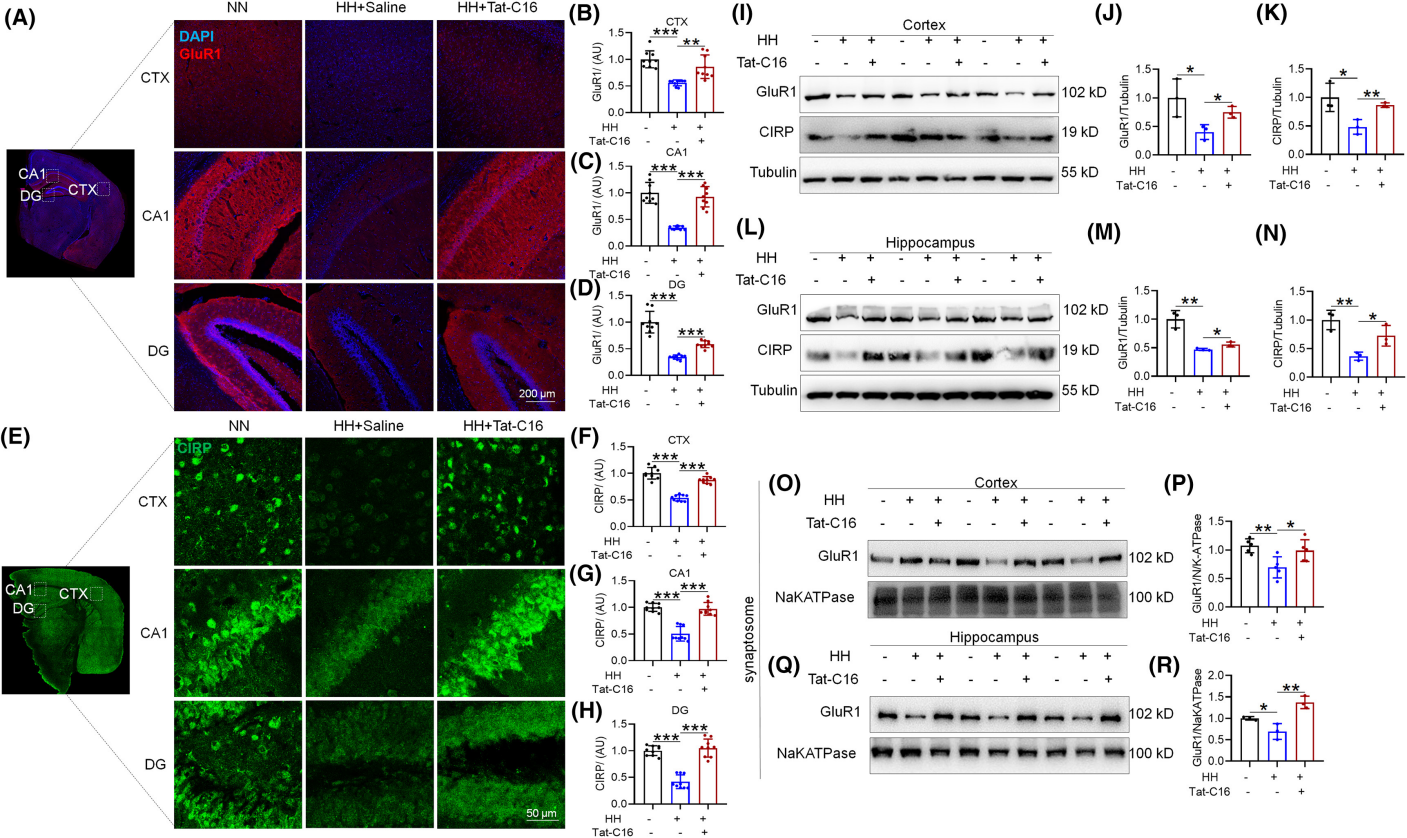
Tat‐C16 peptide mitigates HH‐induced alterations in GluR1 and CIRP within the mouse brain. (A,E) Immunofluorescence showcases the expression and localization of GluR1 and CIRP in the CTX, CA1, and DG following 14 days of HH exposure. (B–D) Quantitative analysis of GluR1 fluorescence intensity across CTX, CA1, and DG regions, indicating changes in synaptic protein distribution. (F–H) Quantitative evaluation of CIRP fluorescence intensity, revealing alterations in protein expression within these brain regions. (I,L) Western blot analyses detail the expression shifts of GluR1 and CIRP in total protein extracts from the cerebral cortex and hippocampus post‐exposure. (J,K, O–Q) Statistical representation of GluR1 and CIRP levels in total protein extracts, highlighting the impact of Tat‐C16 peptide intervention. (P,R) Analysis of GluR1 and CIRP expression in synaptosomes from the cortex and hippocampus, demonstrating the peptide's effect on synaptic components. Significance is marked as *p < 0.05, **p < 0.01, ***p < 0.001 relative to the control group, *n* = 3.

**FIGURE 11:**
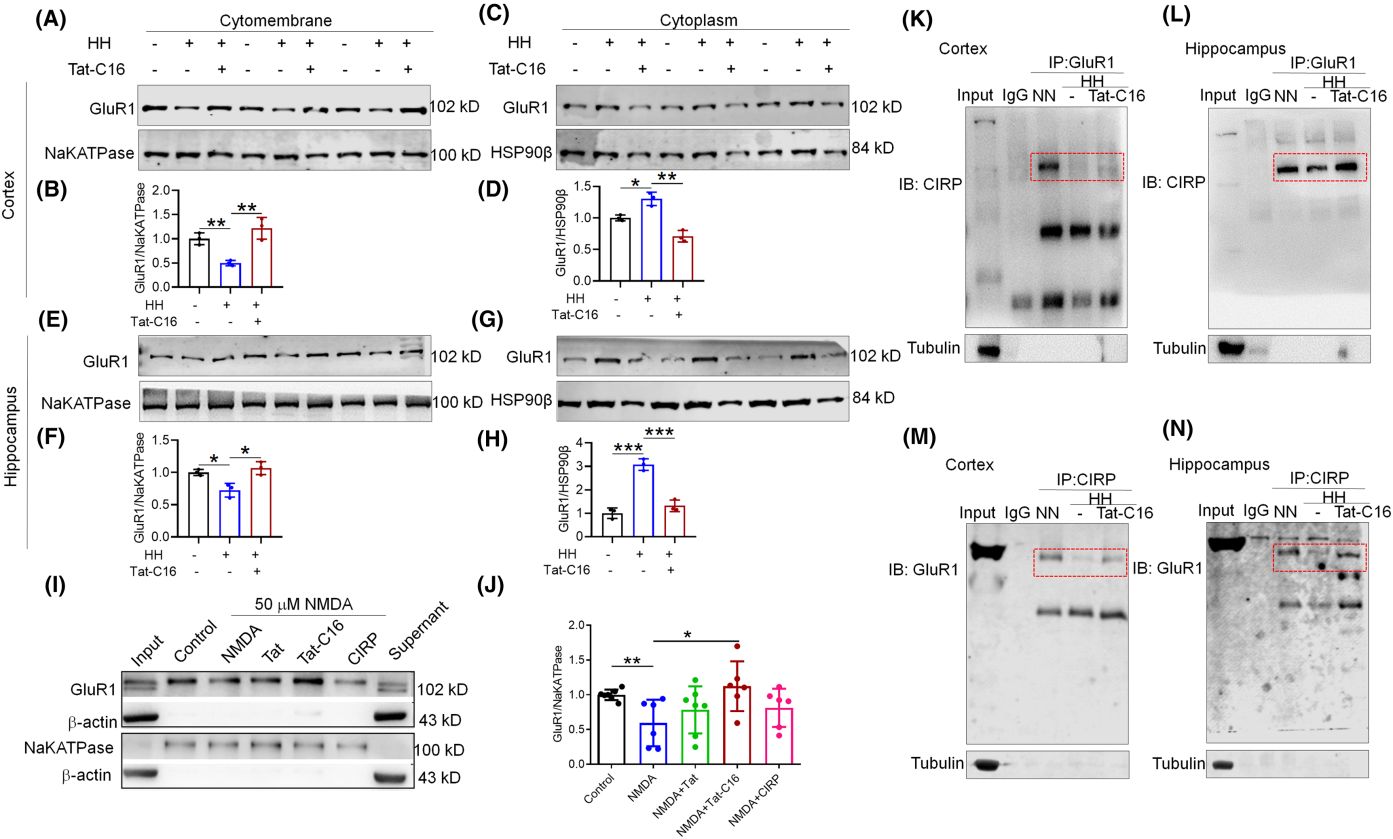
Tat‐C16 peptide modulates GluR1‐CIRP dynamics in mice exposed to HH. (A,E) Western blot (WB) analysis reveals alterations in GluR1 and CIRP protein levels within membrane fractions of the cerebral cortex and hippocampus following 14 days of HH exposure. (C,G) WB analysis shows changes in GluR1 and CIRP levels in cytosolic fractions from these brain regions post‐HH. (B,F) Quantitative data on GluR1 expression in membrane fractions of the cortex and hippocampus post‐HH exposure. (D,H) Statistical analysis of GluR1 expression across both membrane and cytosolic fractions in these areas after HH. (I) WB evaluation of GluR1 presence on neuronal cell membranes. (J) Quantification of GluR1 expression as depicted in panel (A). Co‐IP studies (K,L) assess the interaction dynamics between GluR1 and CIRP in the cortex and hippocampus, with IP targeting GluR1 and IB detecting CIRP. Further Co‐IP analyses (M,N) explore these interactions with IP targeting CIRP and IB identifying GluR1, post‐14 days of HH exposure. Significance indicated as *p < 0.05, **p < 0.01, ***p < 0.001 relative to the control group, with a sample size of *n* = 9.

Upon administering Tat‐C16 at the onset of HH exposure, subsequent MWM assessments indicated a significant preservation of spatial learning and memory. Mice treated with Tat‐C16 exhibited reduced latency to locate the hidden platform, increased accuracy in navigating toward the original platform location, and enhanced spatial memory, as evidenced by their swimming patterns and time spent in the target quadrant (Figure 9A–H). These behavioral improvements were corroborated by morphological analyses, which showed that Tat‐C16 treatment significantly increased dendritic spine and branch numbers, as well as PSD95 protein expression, indicating enhanced synaptic structure and function (Figure 9I–S). Further immunofluorescence and Western blot analyses across various brain regions and cellular fractions revealed that Tat‐C16 not only elevated GluR1 and CIRP expression levels in the cortex and hippocampus but also counteracted the HH‐induced redistribution of GluR1 from neuronal membranes to the cytoplasm (Figures 10A–H and 11A–H). This intervention effectively maintained GluR1 within synaptosomes and neuronal membranes, suggesting a stabilization of synaptic components critical for cognitive processes (Figures 10I–R and 11I,J). Moreover, the peptide's impact extended to the molecular interaction between CIRP and GluR1, where it mitigated the HH‐induced reduction in their binding affinity, further supporting the role of Tat‐C16 in preserving neuronal function and synaptic plasticity under stress conditions (Figure 11K–N). Overall, the Tat‐C16 peptide emerges as a promising therapeutic agent for protecting against cognitive deficits and synaptic dysfunction induced by HH, offering potential for future treatments aimed at mitigating the adverse effects of HA exposure on brain health.

## 4 DISCUSSION

HH, characteristic of HA environments, presents a significant challenge to cognitive integrity and synaptic structure. The reduction in oxygen availability inherent to HH conditions triggers a cascade of cellular responses that compromise synaptic plasticity, a fundamental mechanism underpinning learning and memory.^28^ Previous studies have shown that prolonged exposure to HH leads to alterations in synaptic morphology, including dendritic spine density and synaptic protein expression, which are closely associated with cognitive performance.^8^, ^29^ The synaptic changes observed under HH conditions, such as decreased spine density and altered expression of synaptic proteins like PSD95 and GluR1, directly impact neuronal connectivity and signal transmission.^30^ These morphological alterations reflect the brain's adaptive response to oxygen deprivation but also signify the underlying cause of cognitive impairments observed in individuals exposed to HAs. The correlation between synaptic morphology and cognitive function is well‐documented, with synaptic plasticity serving as the cellular basis for learning and memory processes.^31^ Research indicates that certain aspects of HH‐induced damage can be mitigated or reversed through timely and targeted interventions.^1^, ^4^, ^32^, ^33^ These findings suggest that the detrimental effects of HH on the brain are not entirely permanent and that synaptic function can be at least partially restored through pharmacological intervention. The effectiveness of such interventions likely depends on several factors, including the duration and severity of hypoxia exposure, the timing of the intervention, and the specific mechanisms targeted by the treatment.

Under HH, both GluR1 and CIRP exhibit significant expression changes that are crucial for the synaptic function and plasticity.^17^, ^34^ GluR1, a subunit of AMPA receptors, plays a fundamental role in synaptic transmission and plasticity. Its phosphorylation state, particularly at sites S831 and S845, is essential for modulating synaptic strength and memory processes.^14^ Hypoxic conditions have been shown to disrupt the normal phosphorylation patterns of GluR1, affecting its synaptic incorporation and function.^3^, ^17^, ^19^ CIRP, on the other hand, responds dynamically to hypoxic stress.^34^ Initially upregulated in response to short‐term hypoxic exposure, CIRP expression decreases with prolonged hypoxia.^34^ This protein is involved in RNA binding and stress response, indicating its role in post‐transcriptional regulation under stress conditions.^22^ The decrease in CIRP under extended HH exposure suggests a compromised cellular stress response mechanism, which could contribute to synaptic dysfunction and cognitive decline.

Our findings underscore the critical impact of GluR1 expression, distribution, and phosphorylation on AMPAR and NMDAR activities, highlighting the detrimental effects of hypoxia on synaptic function and neuronal health. In our investigation, we explored the dynamic interplay between CIRP and the GluR1 subunit of AMPARs, with a particular emphasis on the critical phosphorylation sites S831 and S845. These sites are pivotal in modulating synaptic plasticity via long‐term potentiation (LTP) and depression (LTD). Our findings align with Hey‐Kyoung Lee's discovery that synaptic protein phosphorylation serves as a key mediator of LTP/LTD, with specific phosphorylation patterns of GluR1 influencing AMPAR synaptic incorporation.^14^, ^16^ Notably, LTP enhances GluR1 phosphorylation at S831, while subsequent LTP or LTD increases phosphorylation at S845, suggesting a nuanced mechanism linking GluR1 phosphorylation to synaptic AMPAR dynamics. This phosphorylation interplay is crucial, as mutations at these sites have distinct impacts on AMPAR synaptic insertion, thereby influencing synaptic plasticity and cognitive functions. Our study extends these insights by demonstrating that HH disrupts the normal phosphorylation patterns of GluR1, with a notable reduction in CIRP expression exacerbating these effects. Our preliminary analysis, supported by catRAPID predictions, identified potential CIRP binding sites within the GluR1 sequence, encompassing critical phosphorylation/dephosphorylation sites. Under hypoxia, reduced CIRP expression diminishes its interaction with GluR1, affecting synaptic plasticity. Conversely, Tat‐C16 peptide administration enhances CIRP‐GluR1 binding, promoting GluR1 membrane stability and distribution. This interaction likely influences GluR1 dephosphorylation processes, particularly at S845, thereby modulating synaptic plasticity and mitigating learning and memory deficits induced by HH exposure.

The mechanisms by which GluR1 and CIRP influence synaptic plasticity and cognitive function under HH are multifaceted. GluR1's role in synaptic transmission and plasticity is well established, with its phosphorylation state directly affecting AMPAR trafficking to and from the synaptic membrane.^16^ This trafficking is crucial for LTP and LTD, processes underlying learning and memory.^16^, ^17^ The involvement of CIRP in stress response and RNA binding suggests its role in regulating the expression of proteins essential for synaptic function, including GluR1.^35^ Under normoxic conditions, this interaction supports synaptic integrity by ensuring the proper localization and function of GluR1‐containing AMPARs.^17^ However, under hypoxic conditions, the interaction between CIRP and GluR1 is altered. The reduced expression of CIRP leads to a decrease in GluR1 stability on the synaptic membrane, contributing to synaptic dysfunction and cognitive impairments. This altered interaction under HH suggests a disruption in the molecular mechanisms that support synaptic plasticity and stability, highlighting the importance of CIRP in regulating GluR1 under stress conditions.

CIRP plays a pivotal role in the cellular response to stress, exhibiting a dual regulatory function that significantly impacts the expression and interaction of GluR1, a key subunit of AMPA receptors crucial for synaptic transmission and plasticity. This dual role of CIRP, encompassing both transcriptional and post‐transcriptional mechanisms, underscores its importance in maintaining synaptic integrity and neural cell function, particularly under stress conditions such as HH. Firstly, CIRP may interact with GluR1 mRNA, possibly impeding its stabilization or suppressing its translation, subsequently affecting the availability of GluR1 for AMPAR assembly. This process is crucial for the dynamic regulation of synaptic receptors in response to neuronal activity and stress, supporting synaptic plasticity and cognitive functions.^36^, ^37^ Secondly, CIRP interacts with GluR1 at the protein level, possibly influencing its phosphorylation state, trafficking, and membrane localization. This interaction is critical for the proper functioning of AMPARs, affecting synaptic strength and plasticity. Under normal conditions, this dual regulation by CIRP supports the dynamic modulation of synaptic responses, essential for learning and memory processes.^36^, ^38^

Moreover, the phenotypic analysis of *Cirp* KO mice provides invaluable insights into the specific mechanisms.^39^ The absence of *Cirp* leads to cognitive impairment and alterations in dendritic spine morphology. This analysis also sheds light on the impact of *Cirp* deficiency on GluR1 expression and distribution, underlining the critical role of *Cirp* in maintaining normal cognitive function and synaptic structure. *Cirp* KO mice exhibit pronounced cognitive deficits, as evidenced by impaired performance in tasks measuring spatial memory and learning, such as the Morris water maze. These behavioral impairments are paralleled by significant changes in dendritic spine morphology, including reduced spine density and altered spine structure, which are indicative of synaptic dysfunction. The absence of *Cirp* disrupts the normal regulation of synaptic proteins, including GluR1, crucial for synaptic plasticity and effective synaptic transmission.

The Tat‐C16 peptide functions by mimicking the protective and regulatory roles of CIRP, particularly under conditions of stress such as hypoxia.^40^, ^41^ One of the primary mechanisms through which Tat‐C16 peptide reverses cognitive impairment is by stabilizing and enhancing the expression of GluR1 at the synaptic membrane. This stabilization is crucial for maintaining synaptic plasticity, which underlies learning and memory processes. Tat‐C16 peptide achieves this by interacting with the regulatory elements involved in the translocation and trafficking of GluR1 protein. By enhancing the localization of GluR1 to the synaptic membrane, Tat‐C16 peptide facilitates the strengthening of synaptic connections, thereby improving synaptic efficacy and cognitive function. This interaction not only counters the detrimental effects of hypoxia on synaptic plasticity but also promotes the recovery of cognitive abilities impaired by hypoxic conditions. The feasibility of Tat‐C16 peptide as a therapeutic drug hinges on several factors, including its ability to cross the blood–brain barrier (BBB), its stability and bioavailability in the central nervous system (CNS), and its specificity in targeting the pathways involved in hypoxia‐induced cognitive impairment.^42^, ^43^, ^44^ Preliminary studies suggest that the Tat‐C16 peptide, with its small size and the presence of the Tat domain, may possess the ability to cross the BBB effectively, a crucial requirement for any CNS‐active therapeutic agent. Furthermore, the specificity of Tat‐C16 peptide in modulating GluR1 expression and function presents a significant advantage, potentially minimizing off‐target effects and enhancing therapeutic efficacy.

**FIGURE 12:**
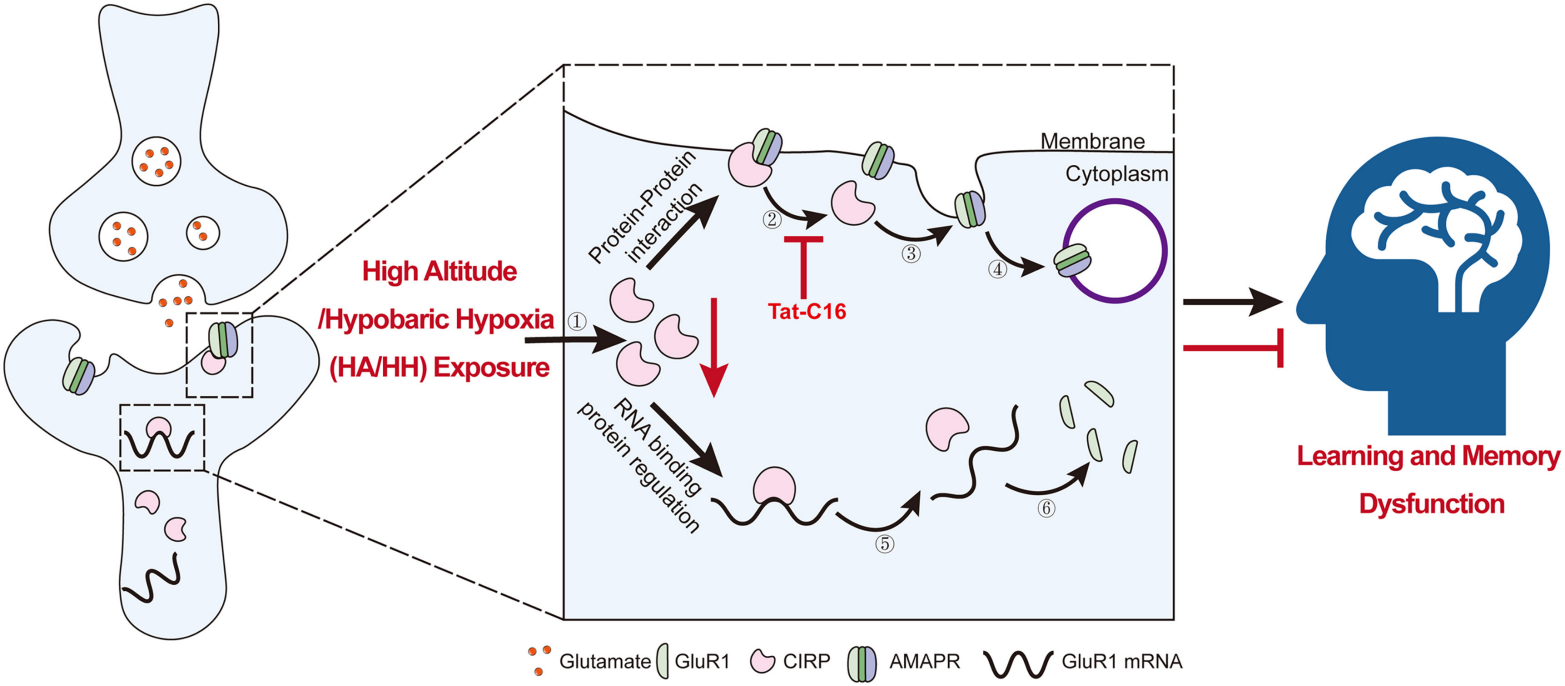
Schematic diagram of the molecular mechanism by which CIRP regulates the expression and distribution of neuronal GluR1 through dual regulatory effects under hypoxic conditions at HA, thereby affecting memory. ① HA/HH exposure leads to a decrease in CIRP expression. ② Reduced binding of CIRP to the GluR1 subunit of AMPAR on the postsynaptic membrane. ③ Endocytosis of AMPAR occurs due to the lack of CIRP stabilization. ④ AMPAR is removed from the membrane through endocytosis. ⑤ The decrease in CIRP expression caused by HA/HH exposure leads to the elimination of its post‐transcriptional inhibition on the *GluR1* gene. ⑥ Increased GluR1 translation promotes an increase in cytoplasmic GluR1 protein.

In summary, this study not only highlights the significant role of CIRP in modulating synaptic plasticity and cognitive functions under HH but also positions it as a promising target for therapeutic interventions aimed at mitigating learning and memory impairments induced by HA exposure (Figure 12). By elucidating the molecular mechanisms by which CIRP influences GluR1 expression and synaptic distribution, we provide a foundational basis for developing strategies to preserve cognitive function in environments characterized by HH. The insights gained from this research contribute to our understanding of the molecular underpinnings of cognitive impairments associated with HH exposure and underscore the potential of targeting the CIRP‐GluR1 axis as a novel approach to enhancing neurological resilience and cognitive performance against hypoxic conditions.

## AUTHOR CONTRIBUTIONS

QQL and GHW conceived, organized, and designed the study. YQG supervised the work. JH, LCY, WHY, DJ, and LJY performed the experiments. JH, LJY and DJF contributed to the analysis of data. QQL and GHW prepared, wrote, and revised the manuscript. All authors contributed to read, and approved the submitted version.

## FUNDING INFORMATION

This work was supported by the National Natural Science Foundation of China (Grants 32271228, 81873924, and 82171190), High‐level Innovation and Entrepreneurship Talents Introduction Program of Jiangsu Province of China, and Nantong Municipal Science and Technology Project (JC22022015). Large Instruments Open Foundation of Nantong University (KFJN2376 and KFJN2444).

## CONFLICT OF INTEREST STATEMENT

The authors have no relevant financial or non‐financial interests to disclose.

## Supporting information


Data S1.

Figure S1.

Figure S2.

Figure S3.

## Data Availability

The file of the unedited images is available as Data S1. Other datasets used and/or analyzed during the current study are available from the corresponding author on reasonable request.

## References

[1] Zhang X , Zhang J . The human brain in a high altitude natural environment: a review. Front Hum Neurosci. 2022;16:915995. 36188182 10.3389/fnhum.2022.915995 PMC9520777

[2] Yang W , Li J , Hu J , et al. Hypobaric hypoxia induces iron mobilization from liver and spleen and increases serum iron via activation of ghrelin/GHSR1a/MAPK signalling pathway in mice. Sci Rep. 2023;13(1):20254. 37985861 10.1038/s41598-023-47596-6 PMC10662372

[3] Della Rocca Y , Fonticoli L , Rajan TS , et al. Hypoxia: molecular pathophysiological mechanisms in human diseases. J Physiol Biochem. 2022;78(4):739‐752. 35870078 10.1007/s13105-022-00912-6 PMC9684243

[4] Taylor AT . High‐altitude illnesses: physiology, risk factors, prevention, and treatment. Rambam Maimonides Med J. 2011;2(1):e0022. 23908794 10.5041/RMMJ.10022 PMC3678789

[5] Skrifvars MB , Sekhon M , Åneman EA . Monitoring and modifying brain oxygenation in patients at risk of hypoxic ischaemic brain injury after cardiac arrest. Crit Care. 2021;25(1):312. 34461973 10.1186/s13054-021-03678-3 PMC8406909

[6] Kumari P , Roy K , Wadhwa M , et al. Fear memory is impaired in hypobaric hypoxia: role of synaptic plasticity and neuro‐modulators in limbic region. Life Sci. 2020;254:117555. 32188570 10.1016/j.lfs.2020.117555

[7] Cunnane SC , Trushina E , Morland C , et al. Brain energy rescue: an emerging therapeutic concept for neurodegenerative disorders of ageing. Nat Rev Drug Discov. 2020;19(9):609‐633. 32709961 10.1038/s41573-020-0072-x PMC7948516

[8] Pun M , Hartmann SE , Furian M , et al. Effect of acute, subacute, and repeated exposure to high altitude (5050 m) on psychomotor vigilance. Front Physiol. 2018;9:677. 29915546 10.3389/fphys.2018.00677 PMC5994420

[9] Maiti P , Muthuraju S , Ilavazhagan G , Singh SB . Hypobaric hypoxia induces dendritic plasticity in cortical and hippocampal pyramidal neurons in rat brain. Behav Brain Res. 2008;189(2):233‐243. 18321600 10.1016/j.bbr.2008.01.007

[10] Abraham WC , Jones OD , Glanzman DL . Is plasticity of synapses the mechanism of long‐term memory storage? Npj Sci Learn. 2019;4(1):9. 31285847 10.1038/s41539-019-0048-y PMC6606636

[11] He Y , Kulasiri D , Samarasinghe S . Modelling bidirectional modulations in synaptic plasticity: a biochemical pathway model to understand the emergence of long term potentiation (LTP) and long term depression (LTD). J Theor Biol. 2016;403:159‐177. 27185535 10.1016/j.jtbi.2016.05.015

[12] Ma S , Zuo Y . Synaptic modifications in learning and memory–a dendritic spine story. Semin Cell Dev Biol. 2022;125:84‐90. 34020876 10.1016/j.semcdb.2021.05.015 PMC9547722

[13] Wang JQ , Arora A , Yang L , et al. Phosphorylation of AMPA receptors: mechanisms and synaptic plasticity. Mol Neurobiol. 2005;32(3):237‐249. 16385140 10.1385/MN:32:3:237

[14] Lee HK , Takamiya K , He K , Song L , Huganir RL . Specific roles of AMPA receptor subunit GluR1 (GluA1) phosphorylation sites in regulating synaptic plasticity in the CA1 region of hippocampus. J Neurophysiol. 2010;103(1):479‐489. 19906877 10.1152/jn.00835.2009 PMC2807233

[15] Lee HK , Kameyama K , Huganir RL , Bear MF . NMDA induces long‐term synaptic depression and dephosphorylation of the GluR1 subunit of AMPA receptors in hippocampus. Neuron. 1998;21(5):1151‐1162. 9856470 10.1016/s0896-6273(00)80632-7

[16] Lee H‐K , Takamiya K , Han J‐S , et al. Phosphorylation of the AMPA receptor GluR1 subunit is required for synaptic plasticity and retention of spatial memory. Cell. 2003;112(5):631‐643. 12628184 10.1016/s0092-8674(03)00122-3

[17] Kopec CD , Real E , Kessels HW , Malinow R . GluR1 links structural and functional plasticity at excitatory synapses. J Neurosci. 2007;27(50):13706‐13718. 18077682 10.1523/JNEUROSCI.3503-07.2007 PMC6673607

[18] Lin CH , Lee EH . JNK1 inhibits GluR1 expression and GluR1‐mediated calcium influx through phosphorylation and stabilization of Hes‐1. J Neurosci. 2012;32(5):1826‐1846. 22302822 10.1523/JNEUROSCI.3380-11.2012 PMC6703358

[19] Du J , Gray NA , Falke C , Yuan P , Szabo S , Manji HK . Structurally dissimilar antimanic agents modulate synaptic plasticity by regulating AMPA glutamate receptor subunit GluR1 synaptic expression. Ann N Y Acad Sci. 2003;1003:378‐380. 14684466 10.1196/annals.1300.031

[20] Liu Y , Xing J . Chronic hypoxia‐induced Cirbp hypermethylation attenuates hypothermic cardioprotection via down‐regulation of ubiquinone biosynthesis. Sci Transl Med. 2019;11(489):eaat8406. 31019028 10.1126/scitranslmed.aat8406

[21] Lujan DA , Ochoa JL , Hartley RS . Cold‐inducible RNA binding protein in cancer and inflammation. Wiley Interdiscip Rev RNA. 2018;9:1‐10. 10.1002/wrna.1462 PMC5886743 29322631

[22] Liao Y , Tong L , Tang L , Wu S . The role of cold‐inducible RNA binding protein in cell stress response. Int J Cancer. 2017;141(11):2164‐2173. 28608439 10.1002/ijc.30833

[23] Qiang XL , Yang WL , Wu RQ , et al. Cold‐inducible RNA‐binding protein (CIRP) triggers inflammatory responses in hemorrhagic shock and sepsis. Nat Med. 2013;19(11):1489‐1495. 24097189 10.1038/nm.3368 PMC3826915

[24] Fang ZP , Wu D , Deng J , et al. An MD2‐perturbing peptide has therapeutic effects in rodent and rhesus monkey models of stroke. Sci Transl Med. 2021;13(597):eabb6716. 34108252 10.1126/scitranslmed.abb6716

[25] Zuo WQ , Zhao JS , Zhang JM , et al. MD2 contributes to the pathogenesis of perioperative neurocognitive disorder via the regulation of alpha 5GABA(a) receptors in aged mice. J Neuroinflammation. 2021;18(1):204. 34530841 10.1186/s12974-021-02246-4 PMC8444589

[26] Su R , Jia S , Zhang N , et al. The effects of long‐term high‐altitude exposure on cognition: a meta‐analysis. Neurosci Biobehav Rev. 2024;161:105682. 38642865 10.1016/j.neubiorev.2024.105682

[27] Yang WP , Li MQ , Ding J , et al. High‐altitude hypoxia exposure inhibits erythrophagocytosis by inducing macrophage ferroptosis in the spleen. Elife. 2024;12:RP87496. 38629942 10.7554/eLife.87496 PMC11023697

[28] Neves G , Cooke SF , Bliss TVP . Synaptic plasticity, memory and the hippocampus: a neural network approach to causality. Nat Rev Neurosci. 2008;9(1):65‐75. 18094707 10.1038/nrn2303

[29] Ji W , Zhang Y , Ge RL , Wan Y , Liu J . NMDA receptor‐mediated excitotoxicity is involved in neuronal apoptosis and cognitive impairment induced by chronic hypobaric hypoxia exposure at high altitude. High Alt Med Biol. 2021;22(1):45‐57. 33252277 10.1089/ham.2020.0127

[30] Lepeta K , Lourenco MV , Schweitzer BC , et al. Synaptopathies: synaptic dysfunction in neurological disorders‐a review from students to students. J Neurochem. 2016;138(6):785‐805. 27333343 10.1111/jnc.13713 PMC5095804

[31] Yang Y , Lu J , Zuo Y . Changes of synaptic structures associated with learning, memory and diseases. Brain Sci Adv. 2018;4(2):99‐117.

[32] Virolainen SJ , VonHandorf A , Viel KCMF , Weirauch MT , Kottyan LC . Gene–environment interactions and their impact on human health. Genes Immun. 2023;24(1):1‐11. 36585519 10.1038/s41435-022-00192-6 PMC9801363

[33] Kushwah N , Jain V , Dheer A , Kumar R , Prasad D , Khan N . Hypobaric hypoxia‐induced learning and memory impairment: elucidating the role of small conductance Ca(2+)‐activated K(+) channels. Neuroscience. 2018;388:418‐429. 30048783 10.1016/j.neuroscience.2018.07.026

[34] Zhou Y , Lu H , Liu Y , et al. Cirbp‐PSD95 axis protects against hypobaric hypoxia‐induced aberrant morphology of hippocampal dendritic spines and cognitive deficits. Mol Brain. 2021;14(1):129. 34419133 10.1186/s13041-021-00827-1 PMC8379783

[35] Xie D , Geng L , Xiong K , et al. Cold‐inducible RNA‐binding protein prevents an excessive heart rate response to stress by targeting phosphodiesterase. Circ Res. 2020;126(12):1706‐1720. 32212953 10.1161/CIRCRESAHA.119.316322

[36] Li X‐R , Cheng X , Sun J , et al. Acetylation‐dependent glutamate receptor GluR signalosome formation for STAT3 activation in both transcriptional and metabolism regulation. Cell Death Dis. 2021;7(1):11. 10.1038/s41420-020-00389-6 PMC7809112 33446662

[37] Corre M , Lebreton A . Regulation of cold‐inducible RNA‐binding protein (CIRBP) in response to cellular stresses. Biochimie. 2024;217:3‐9. 37037339 10.1016/j.biochi.2023.04.003

[38] Tavalin SJ , Colledge M , Hell JW , Langeberg LK , Huganir RL , Scott JD . Regulation of GluR1 by the A‐kinase anchoring protein 79 (AKAP79) signaling complex shares properties with long‐term depression. J Neurosci. 2002;22(8):3044‐3051. 11943807 10.1523/JNEUROSCI.22-08-03044.2002 PMC6757527

[39] Goossens EAC , Zhang L , de Vries MR , Jukema JW , Quax PHA , Nossent AY . Cold‐inducible RNA‐binding protein but not its antisense lncRNA is a direct negative regulator of angiogenesis in vitro and in vivo via regulation of the 14q32 angiomiRs‐microRNA‐329‐3p and microRNA‐495‐3p. Int J Mol Sci. 2021;22(23):12678. 34884485 10.3390/ijms222312678 PMC8657689

[40] Vivès E , Richard JP , Rispal C , Lebleu B . TAT peptide internalization: seeking the mechanism of entry. Curr Protein Pept Sci. 2003;4(2):125‐132. 12678851 10.2174/1389203033487306

[41] Vives E . Cellular uptake [correction of utake] of the tat peptide: an endocytosis mechanism following ionic interactions. J Mol Recognit. 2003;16(5):265‐271. 14523939 10.1002/jmr.636

[42] Bingor A , Haham T , Thornton C , Stern‐Bach Y , Yaka R . Zeta Inhibitory Peptide attenuates learning and memory by inducing NO‐mediated downregulation of AMPA receptors. Nat Comm. 2020;11(1):3688. 10.1038/s41467-020-17484-y PMC7378180 32703948

[43] Richard JP , Melikov K , Vives E , et al. Cell‐penetrating peptides. A reevaluation of the mechanism of cellular uptake. J Biol Chem. 2003;278(1):585‐590. 12411431 10.1074/jbc.M209548200

[44] Wu Y , Angelova A . Recent uses of lipid nanoparticles, cell‐penetrating and bioactive peptides for the development of brain‐targeted nanomedicines against neurodegenerative disorders. Nanomaterials, (Basel). 2023;13(23):3004. 38063700 10.3390/nano13233004 PMC10708303

