# Supplementary material for: Decreased cold‐inducible RNA‐binding protein (CIRP) binding to GluRl on neuronal membranes mediates memory impairment resulting from prolonged hypobaric hypoxia exposure
Source: CNS Neurosci Ther. 2024 Sep 24;30(9):e70059. doi: 10.1111/cns.70059 (PMC11420629; doi:10.1111/cns.70059)

Blots identification of CIRP

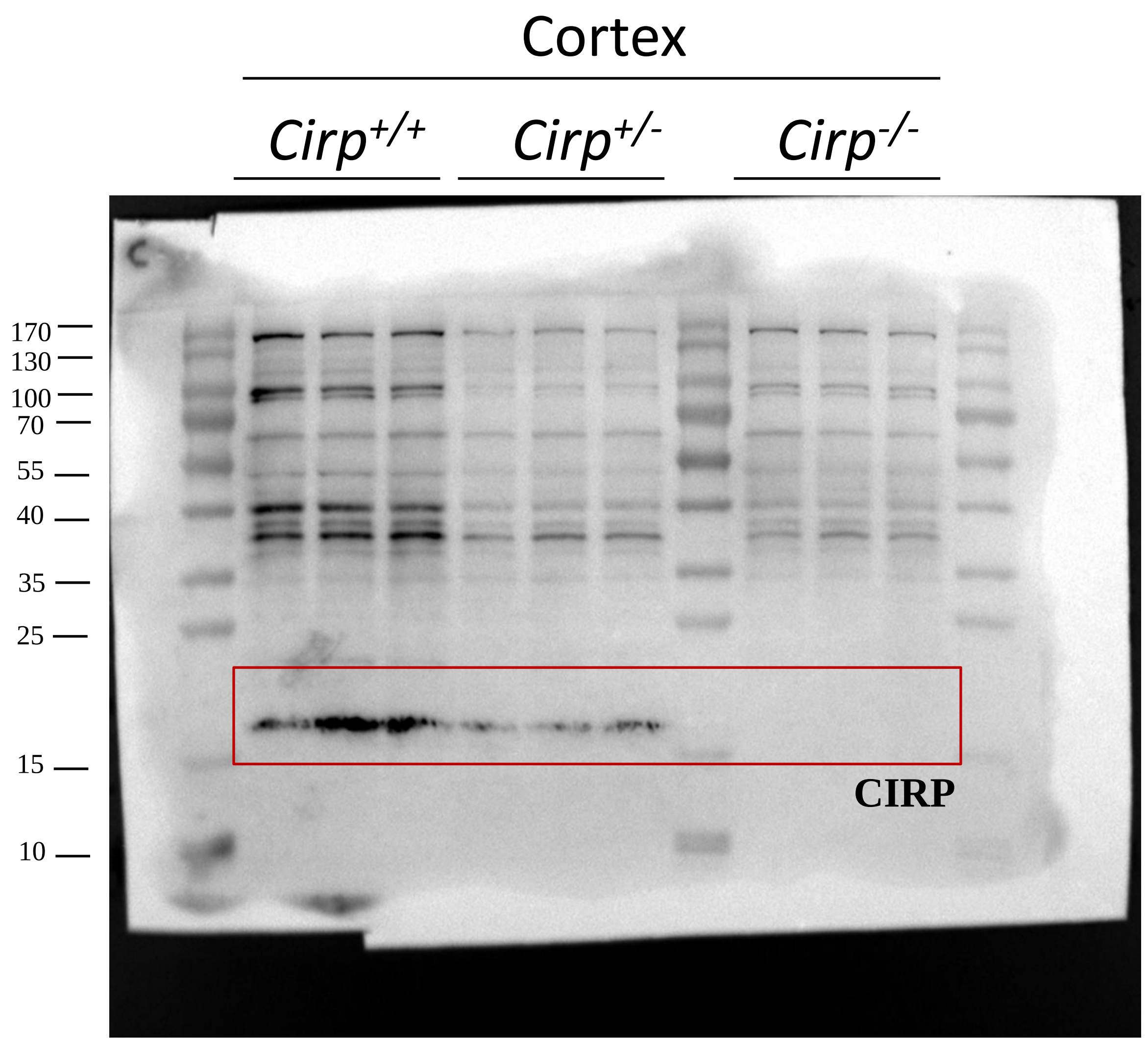

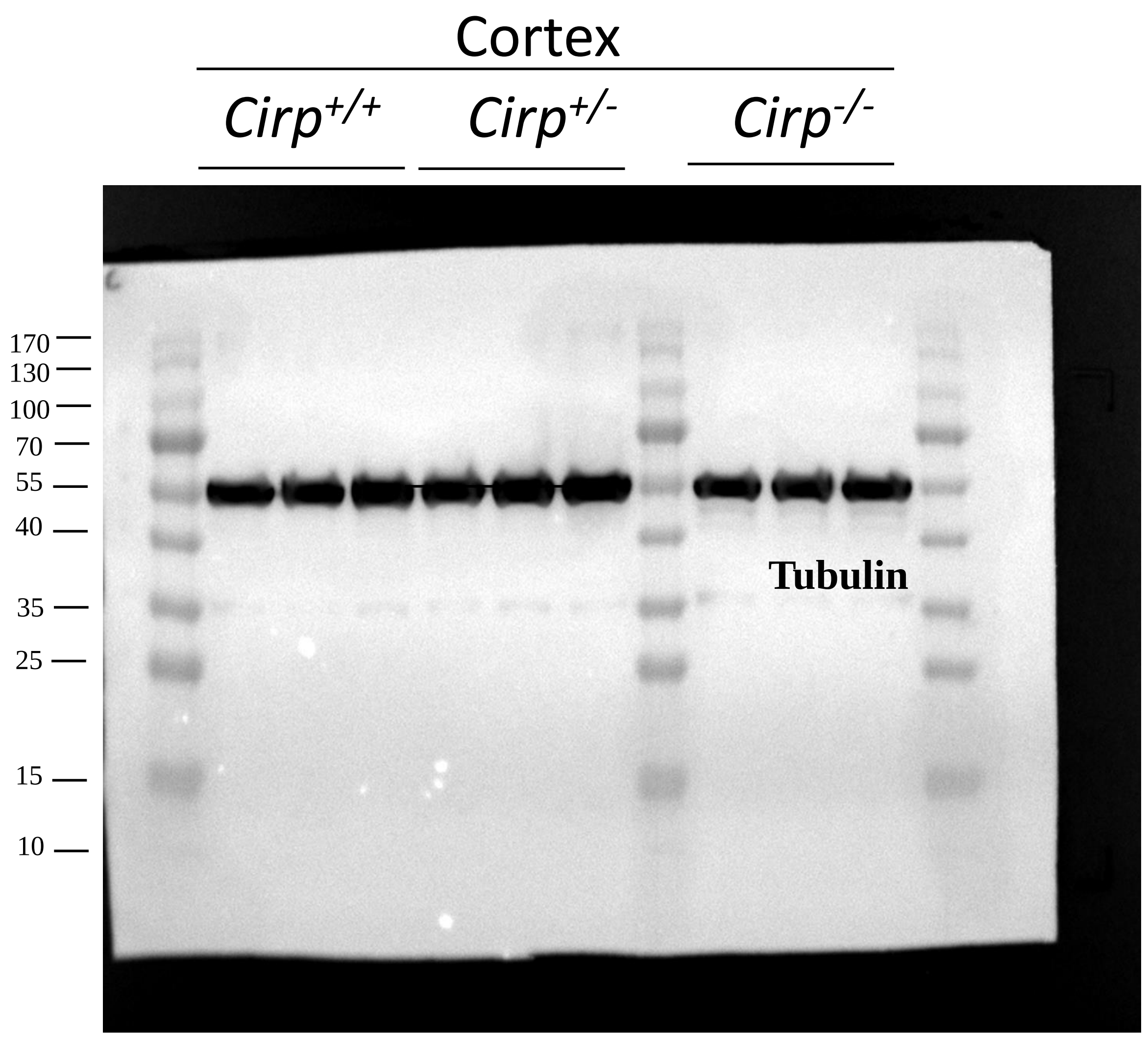

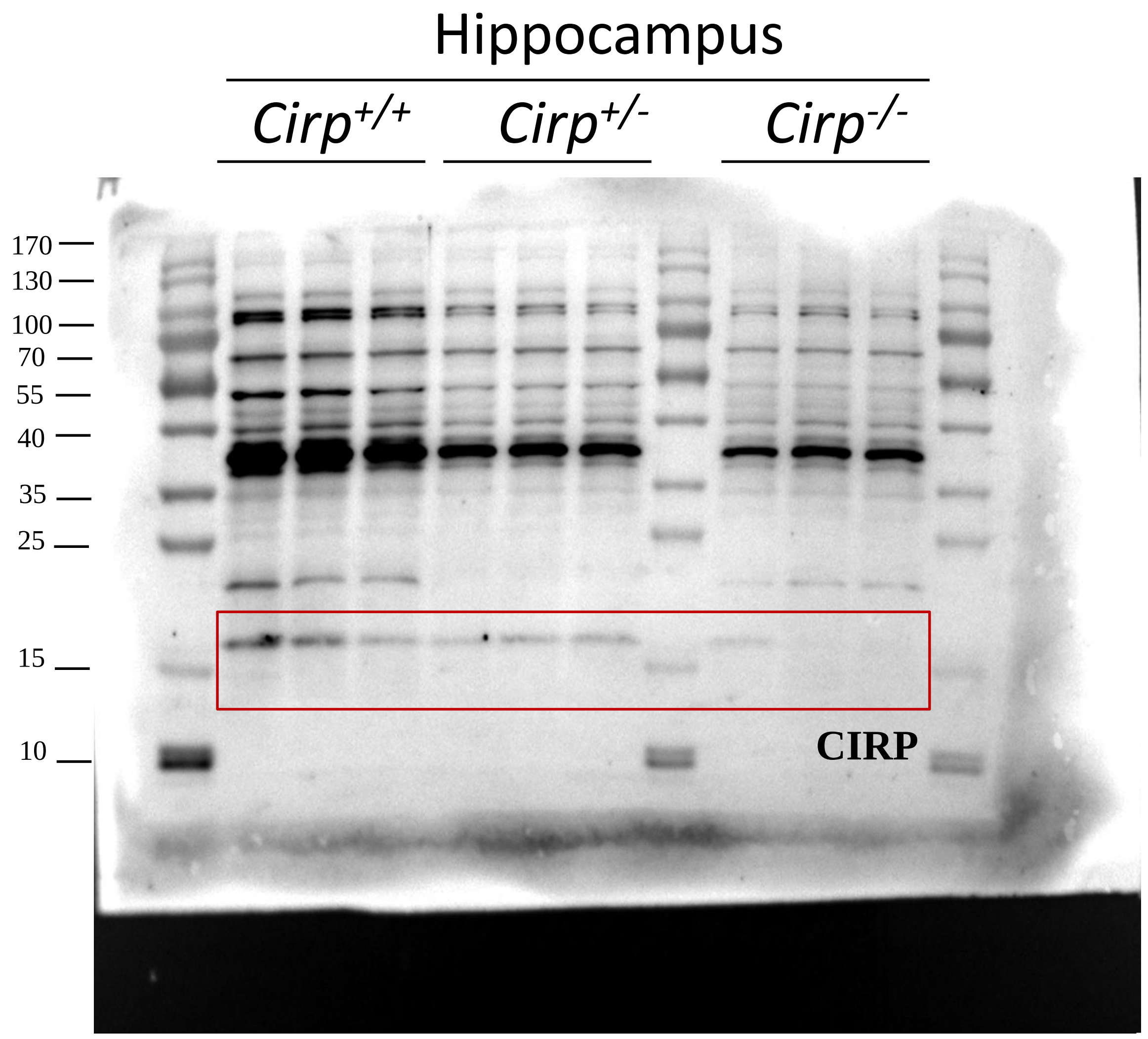

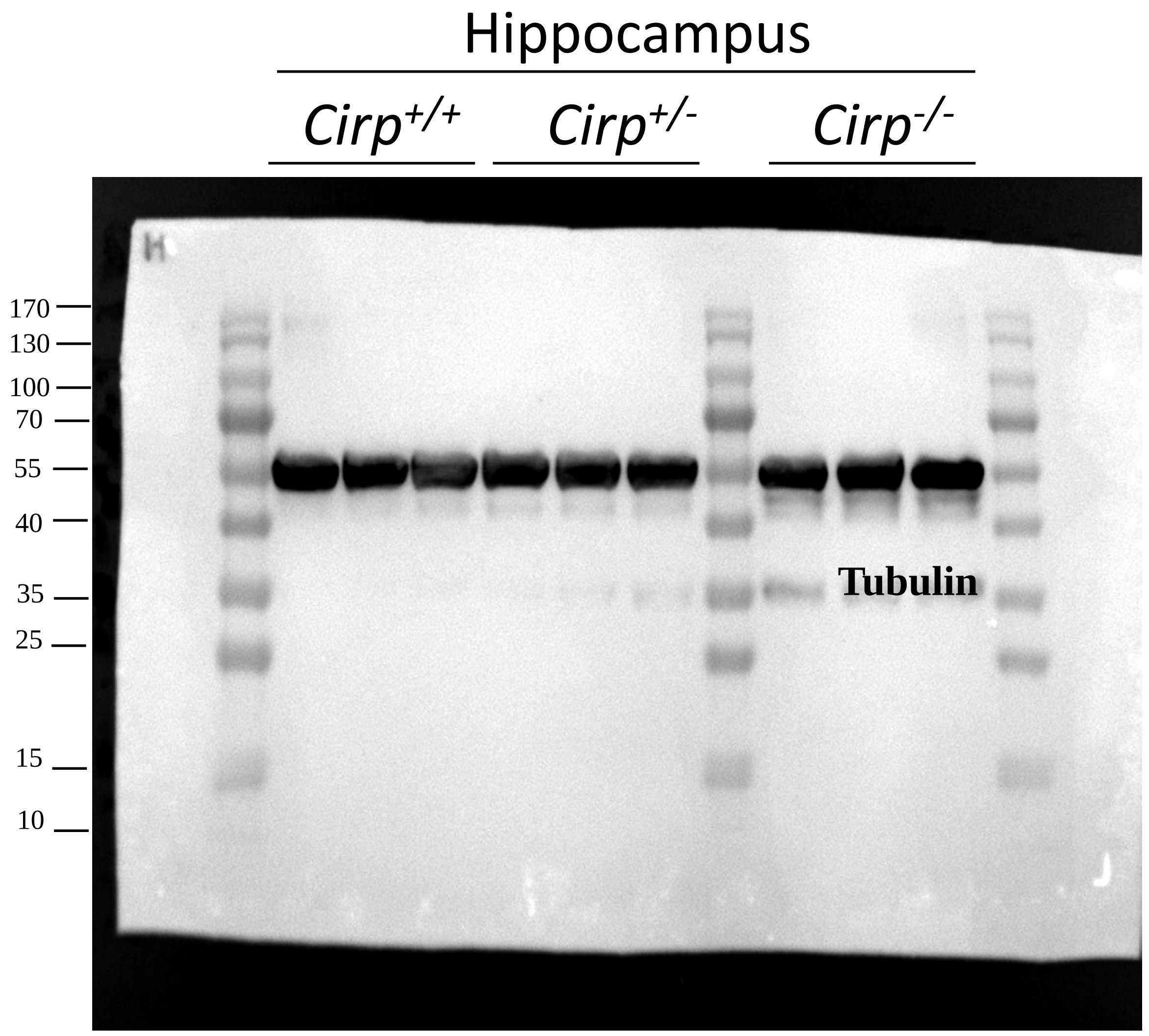

Blots identification of other proteins

Cortex

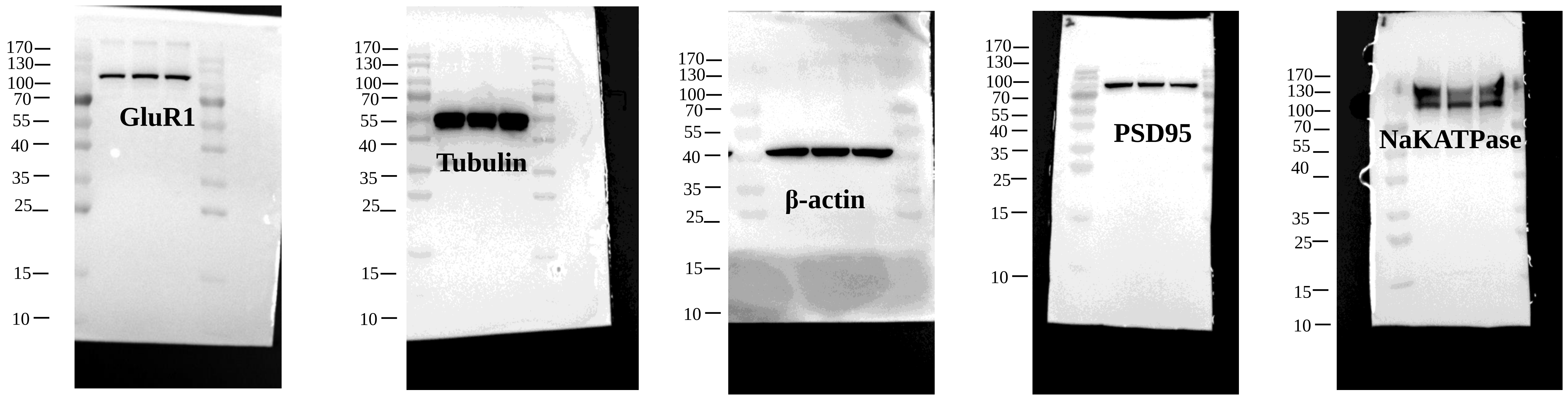

Hippocampus

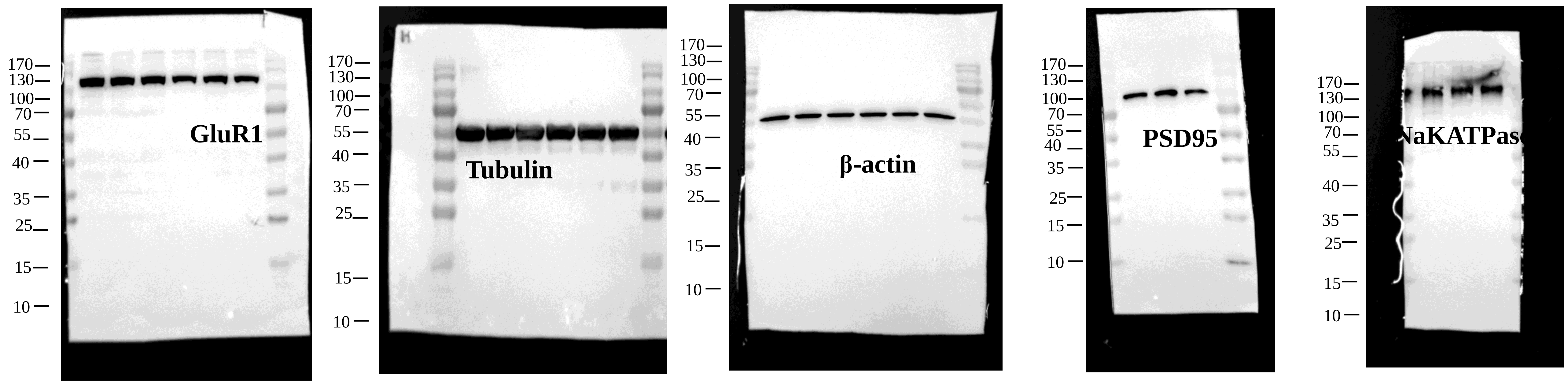

Western Blots in Figure 3

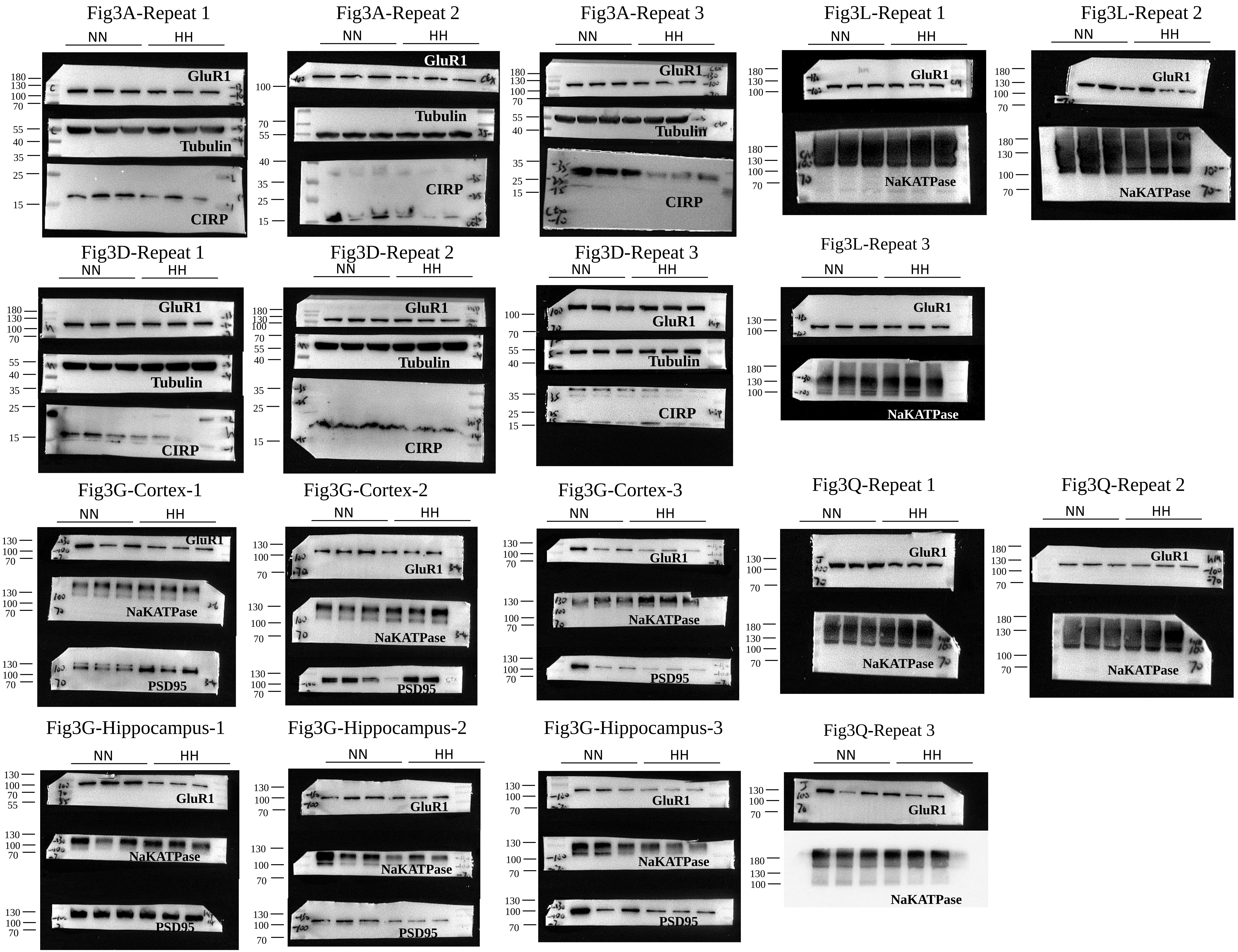

Western Blots in Figure 3

Fig3N-Repeat 1

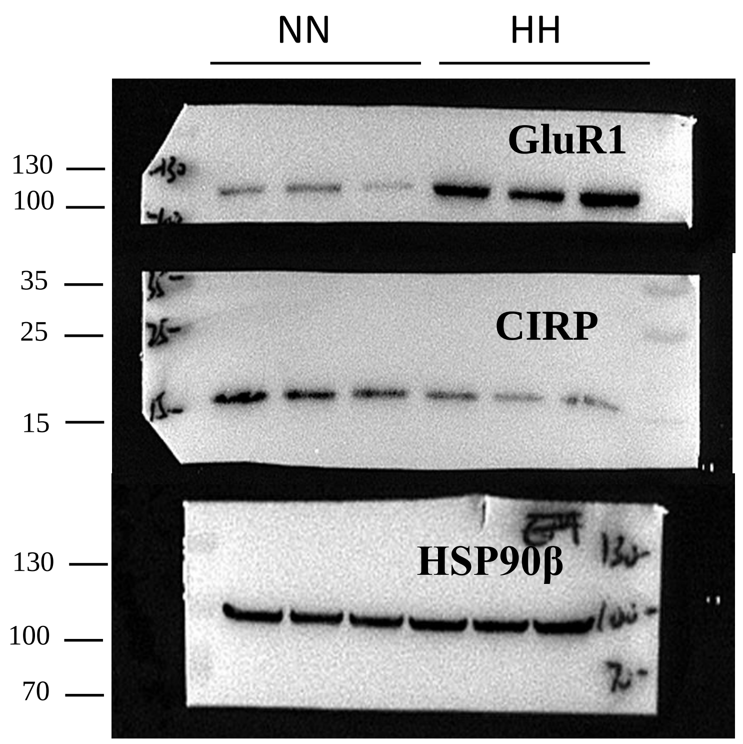

Fig3N-Repeat 2

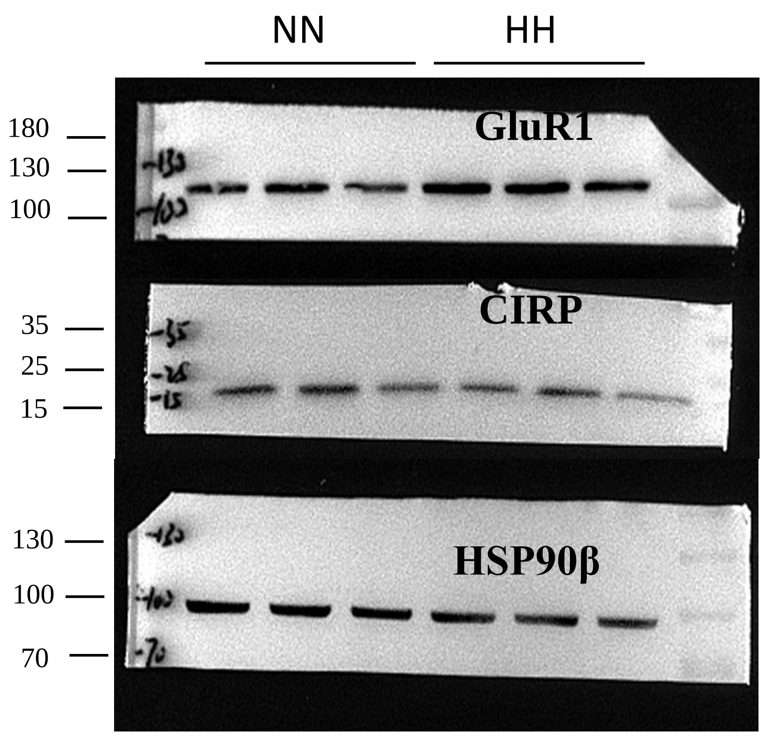

Fig3N-Repeat 3

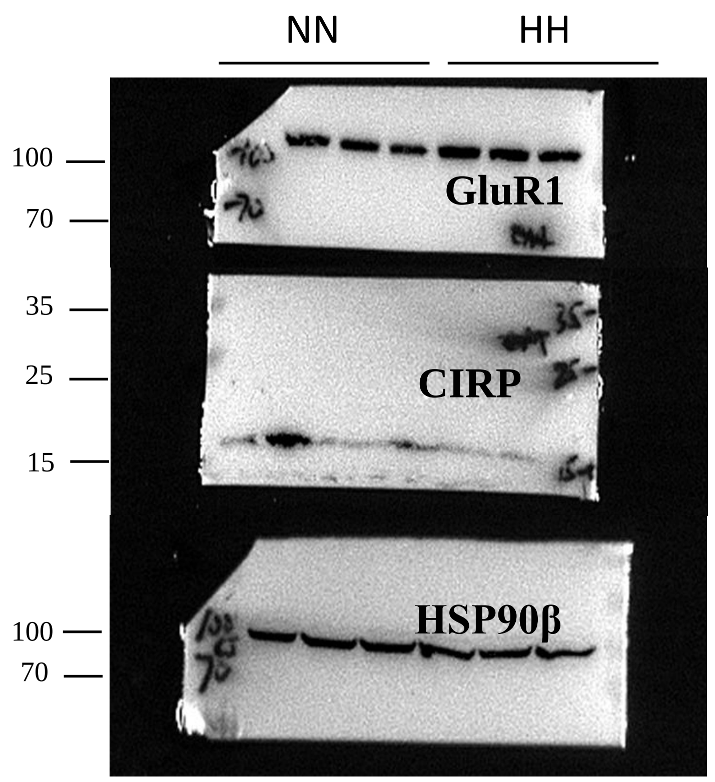

Fig3S-Repeat 1

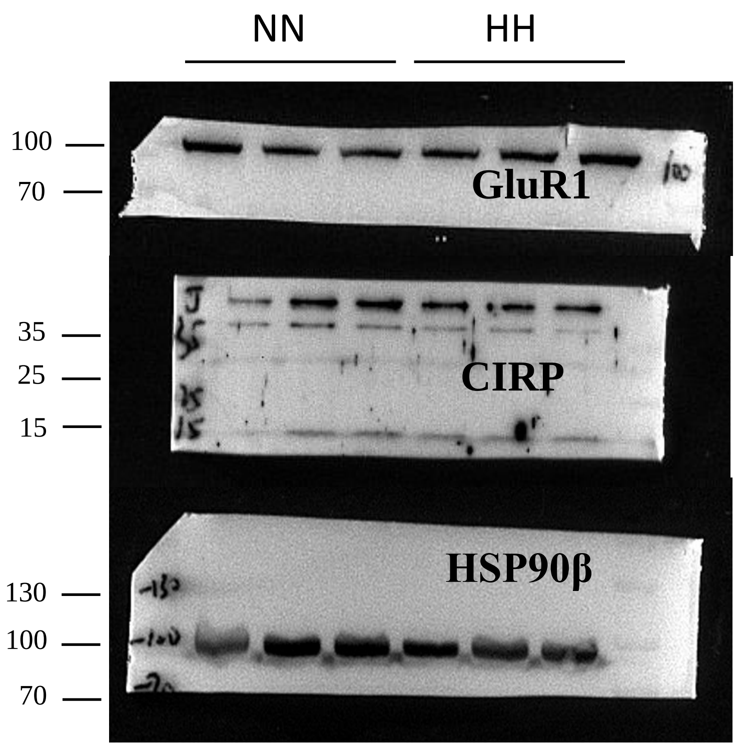

Fig3S-Repeat 1

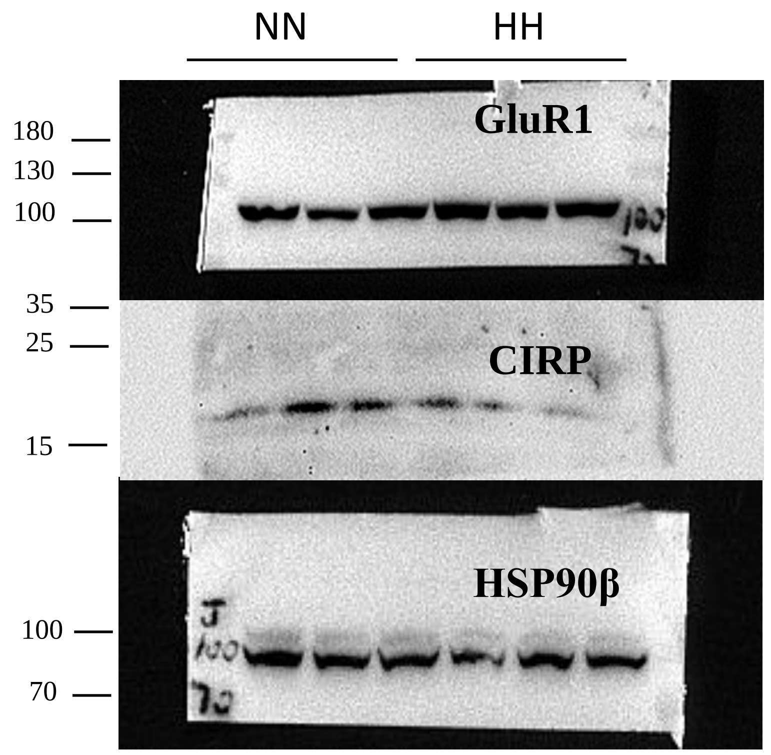

Fig3S-Repeat 3

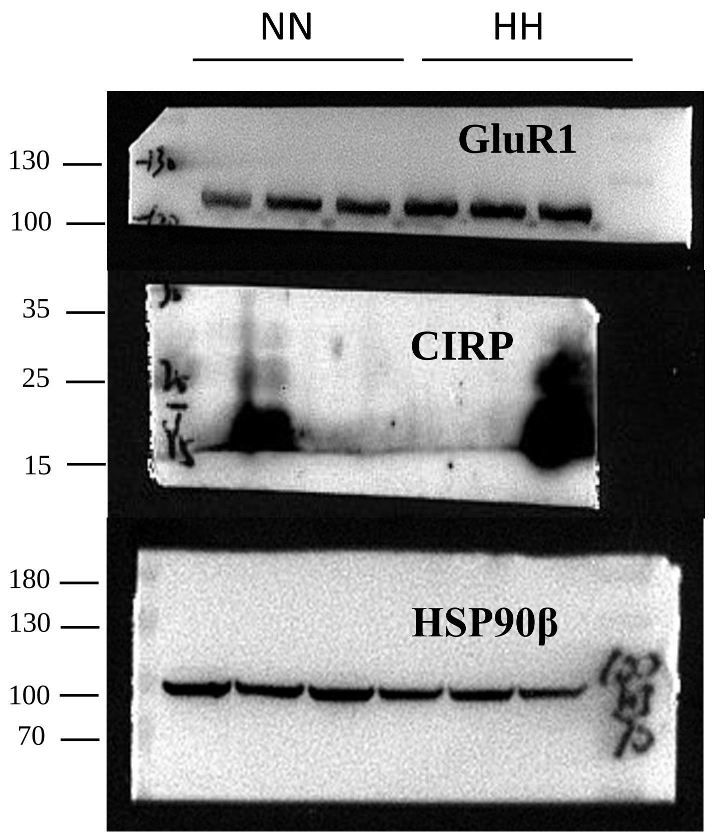

Western Blots in Figure 4

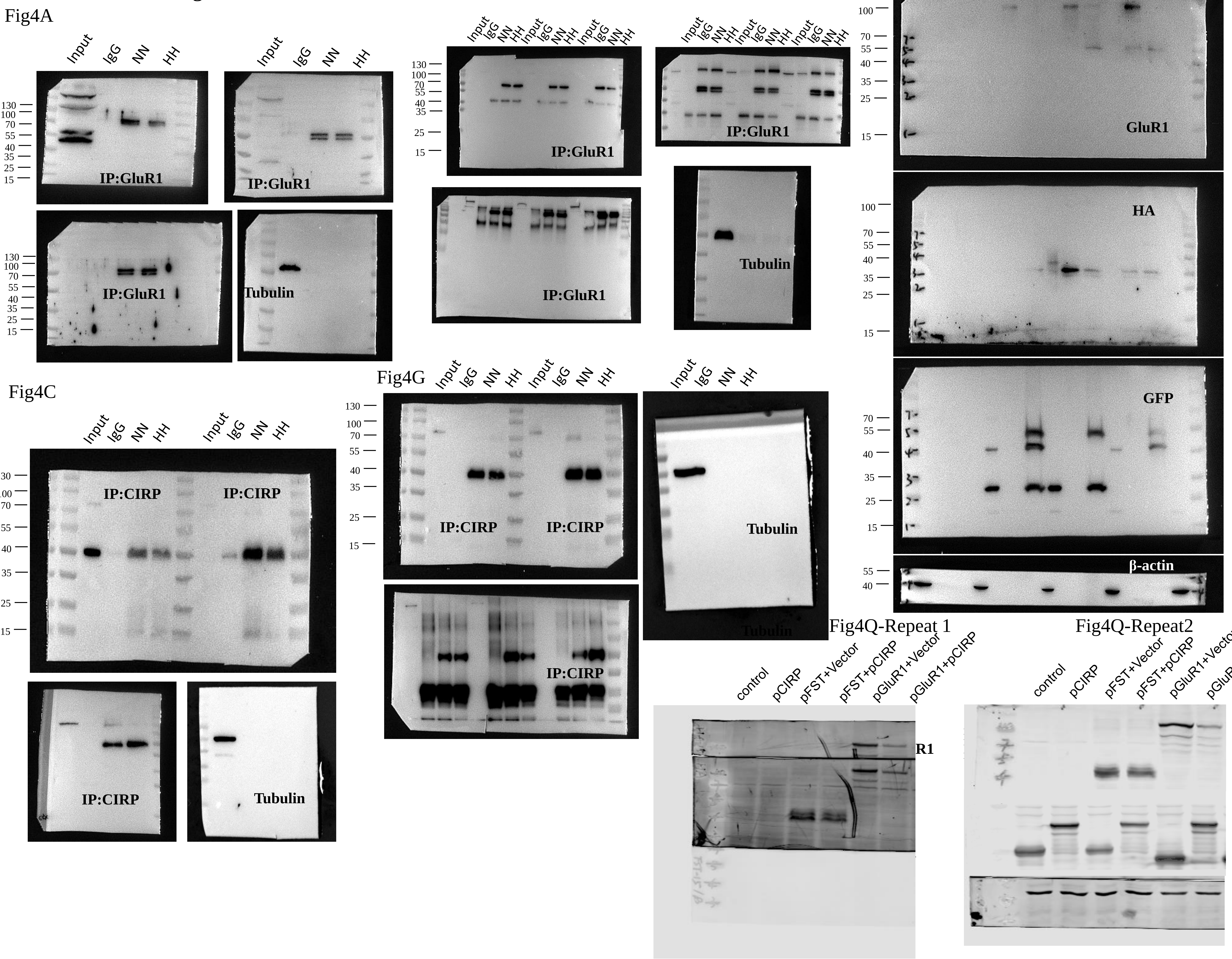

Western Blots in Figure 6

Fig6L-Repeat 1

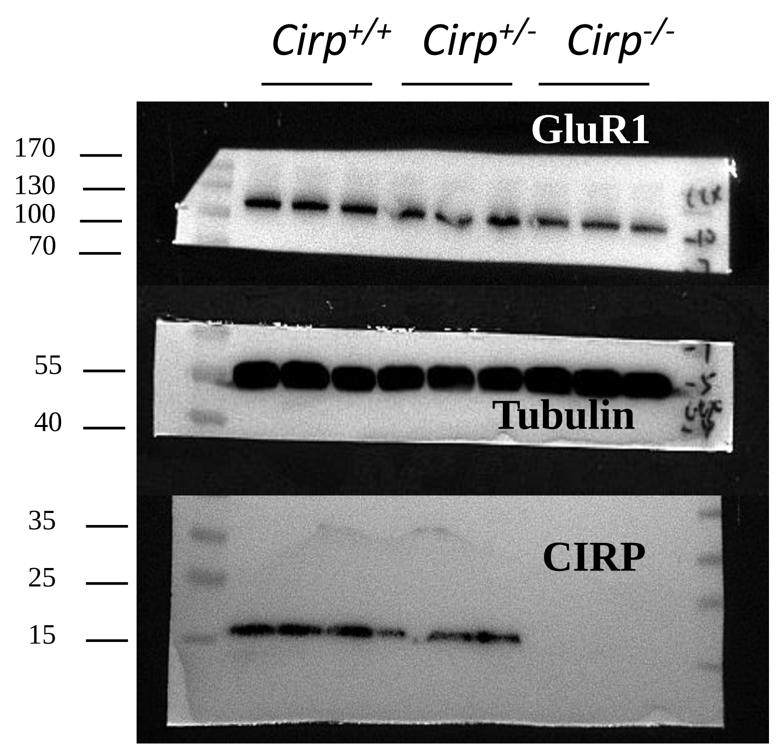

Fig6L-Repeat 2

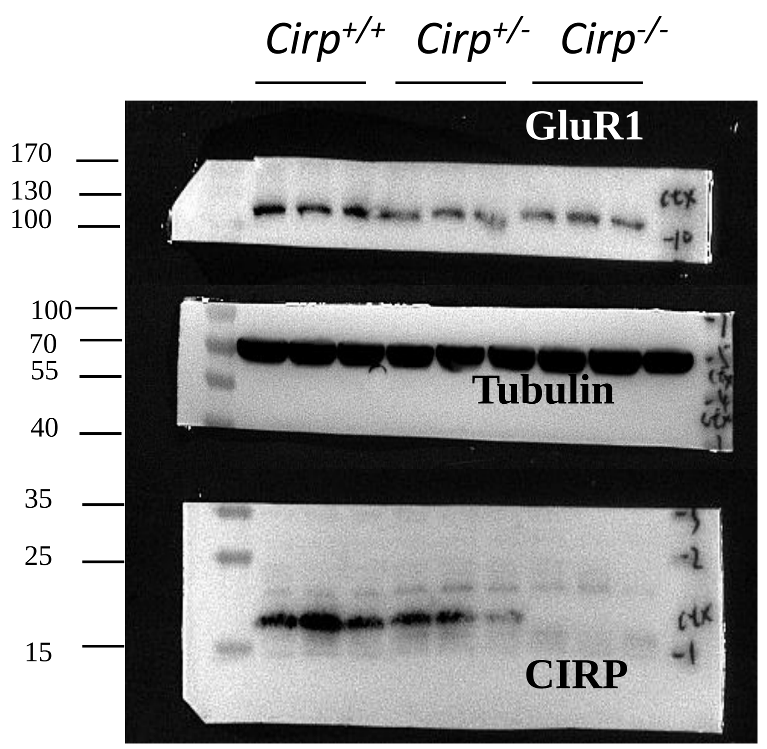

Fig6L-Repeat 3

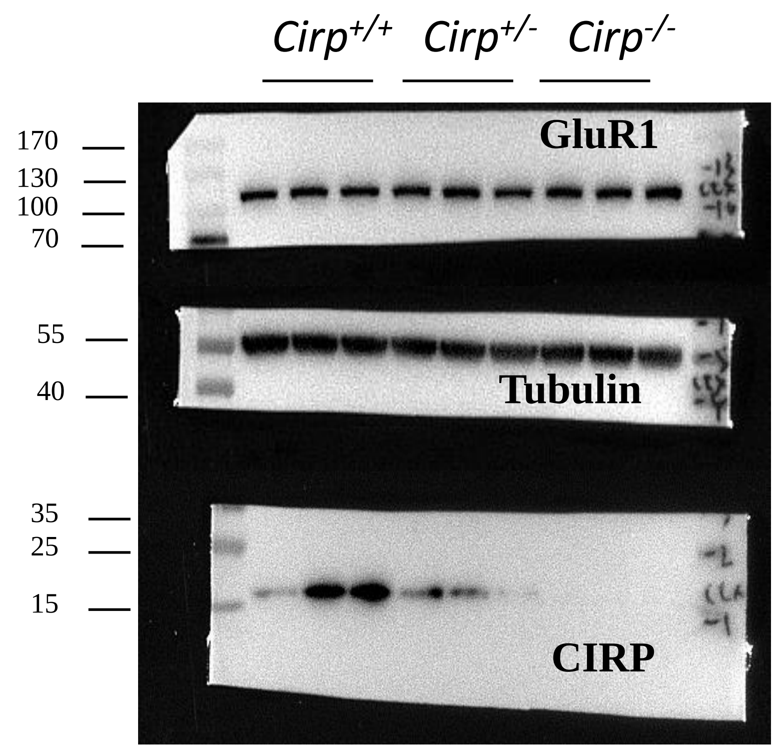

Fig6O-Repeat 1

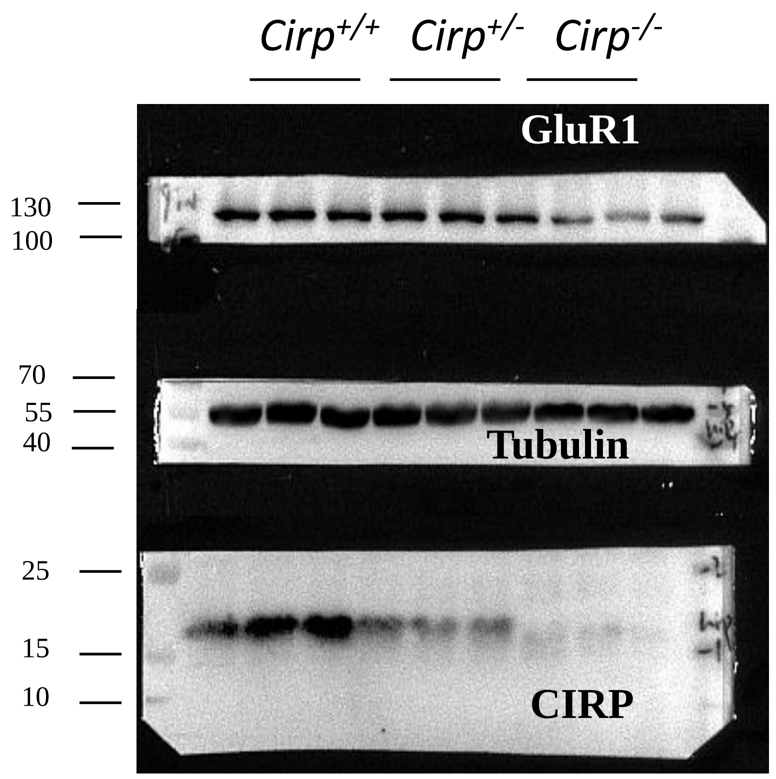

Fig6O-Repeat 2

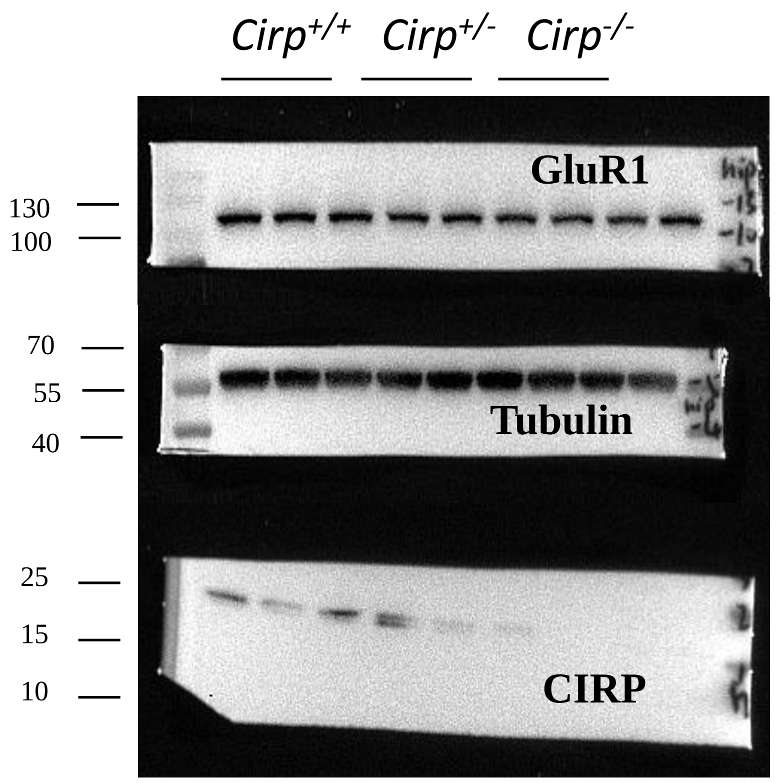

Fig6O-Repeat 3

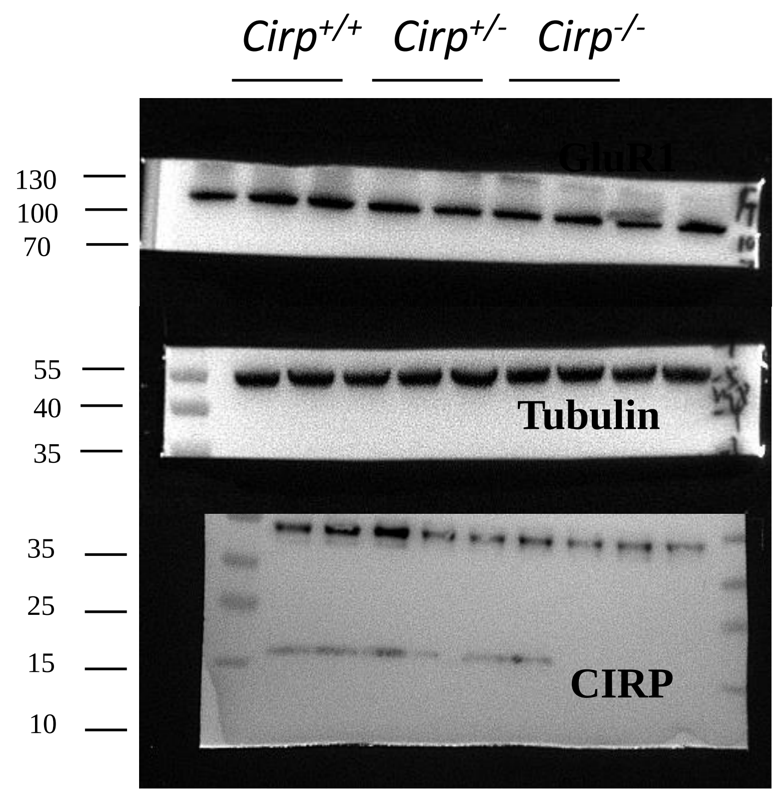

Western Blots in Figure 7

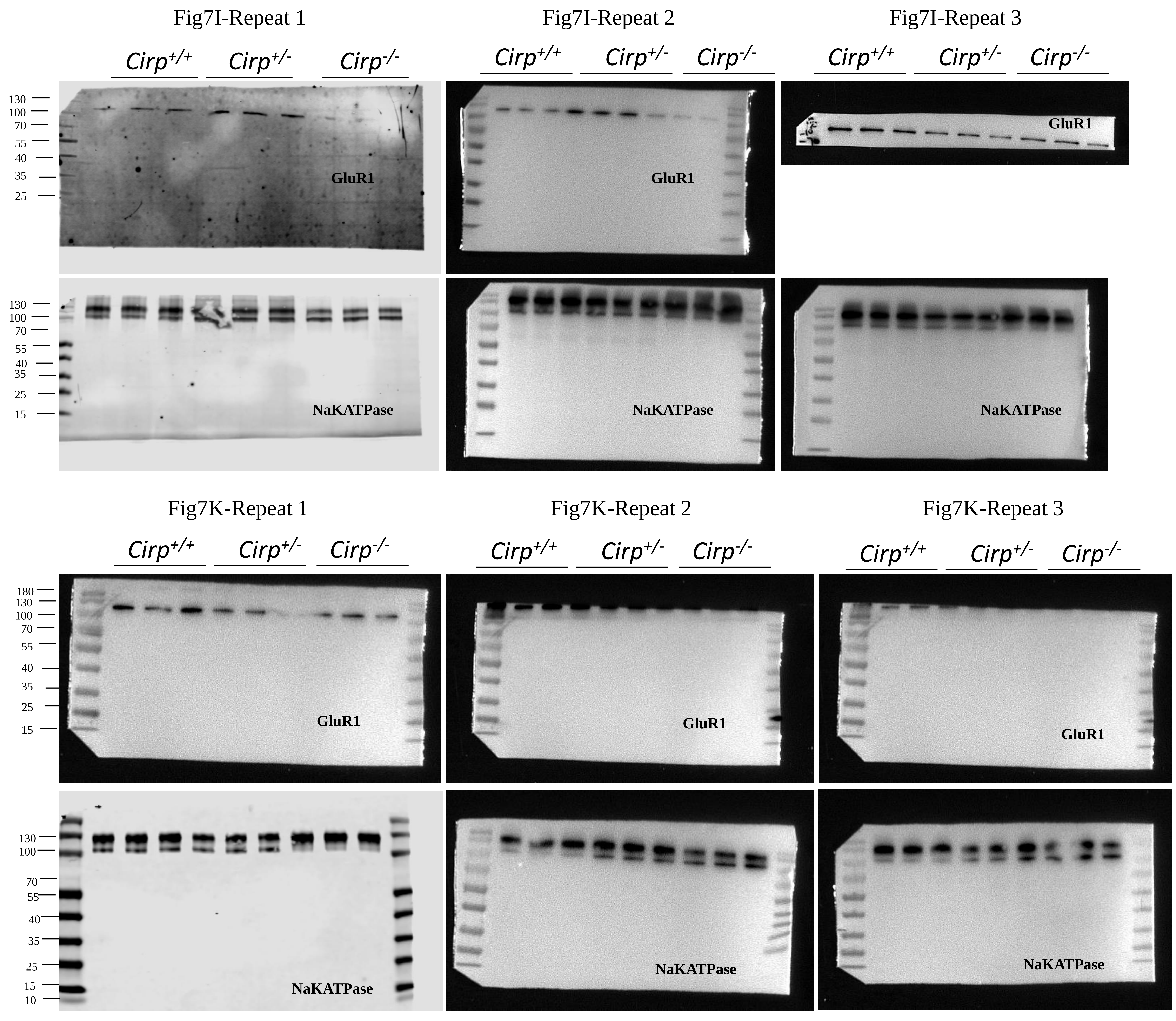

Western Blots in Figure 8

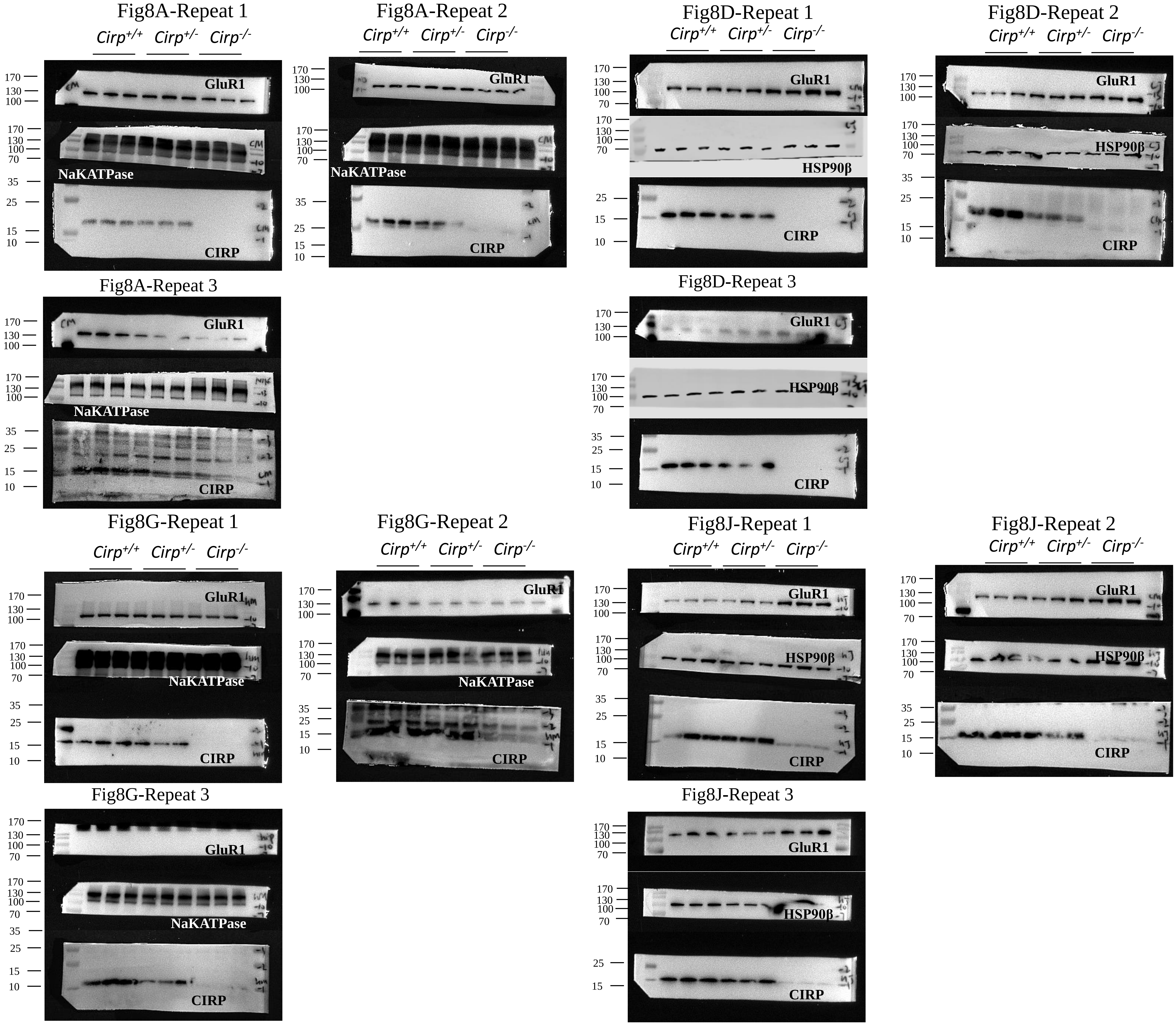

Western Blots in Figure 8

Fig8M

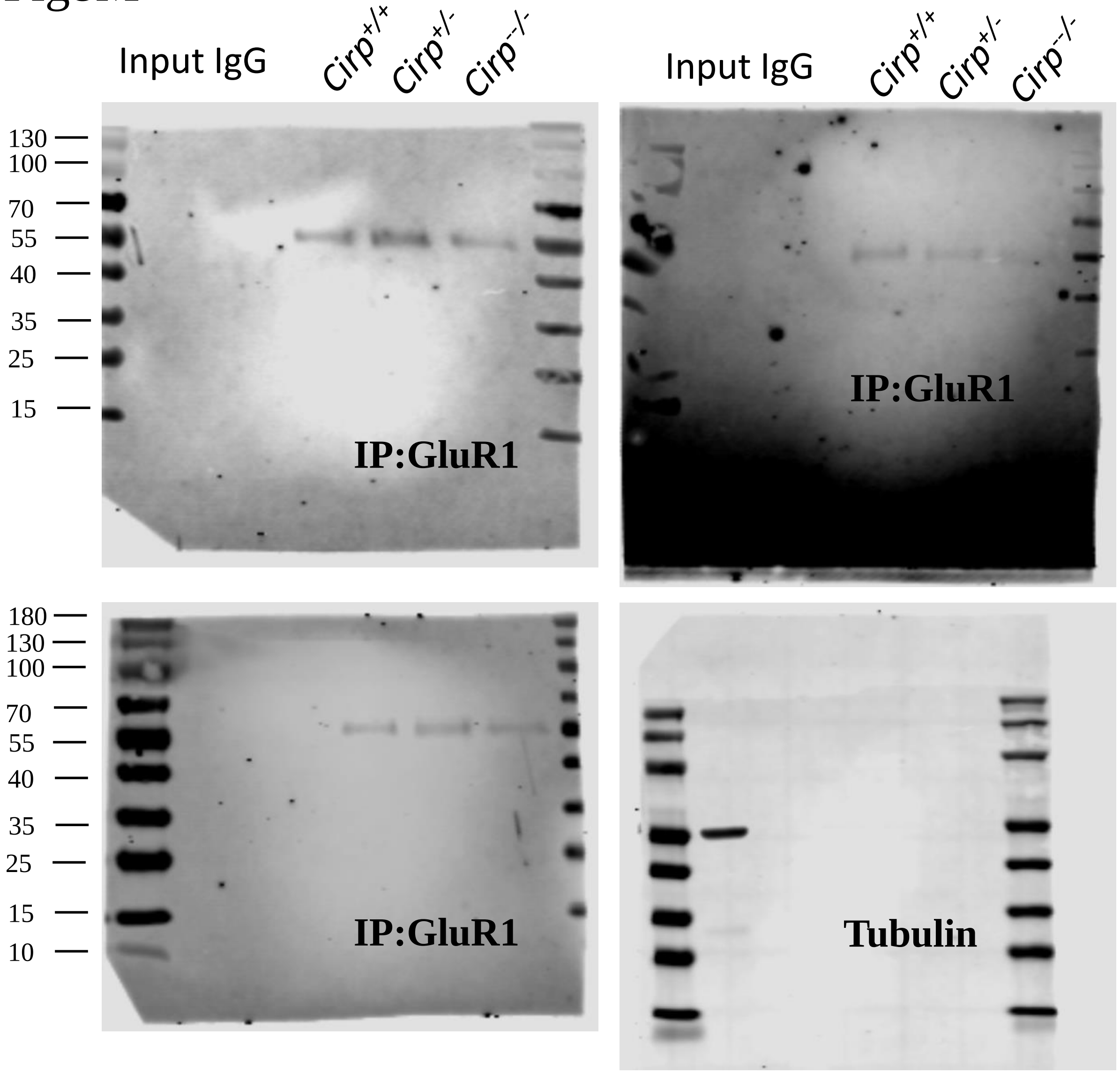

Fig8N

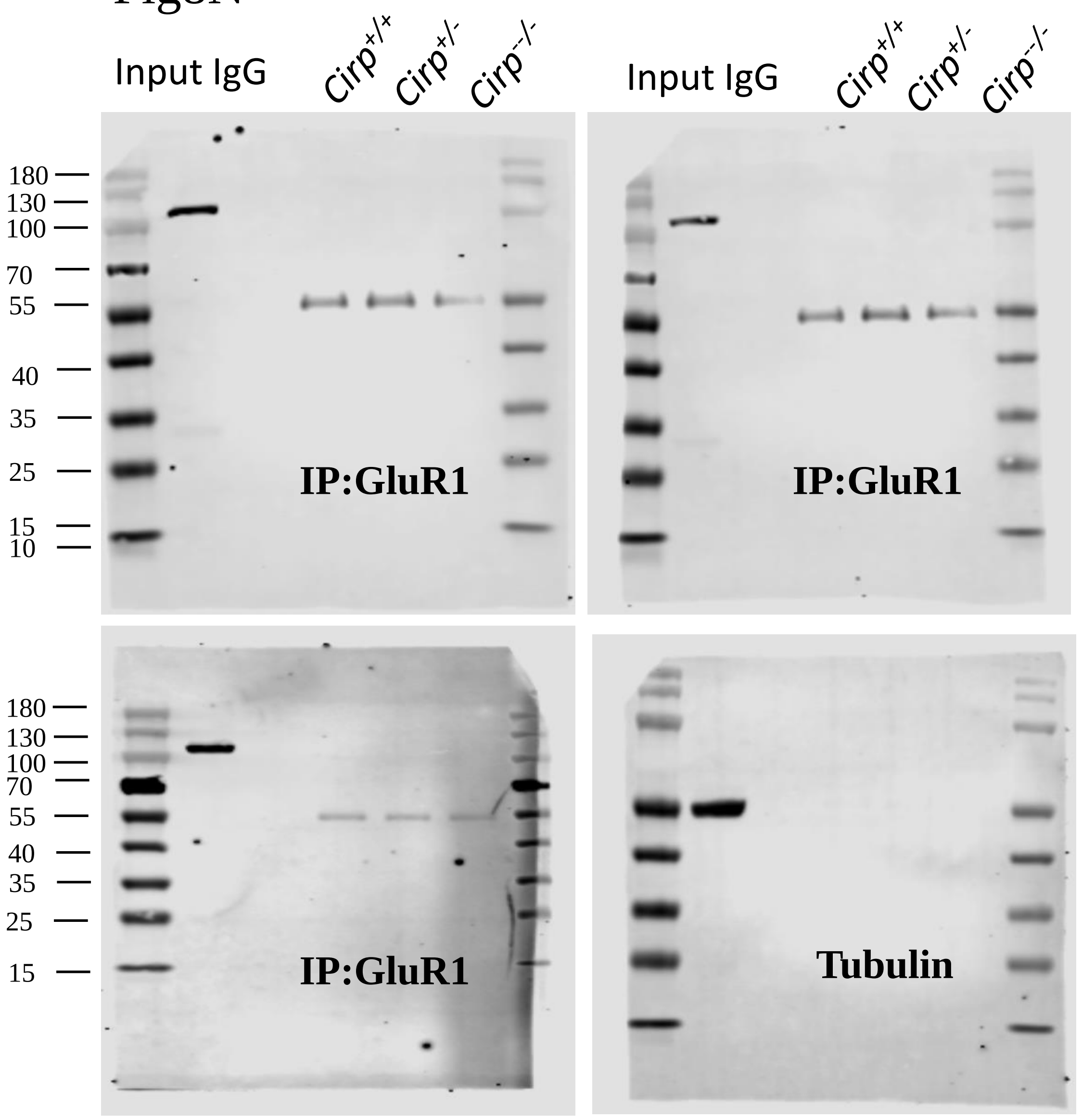

Fig8O

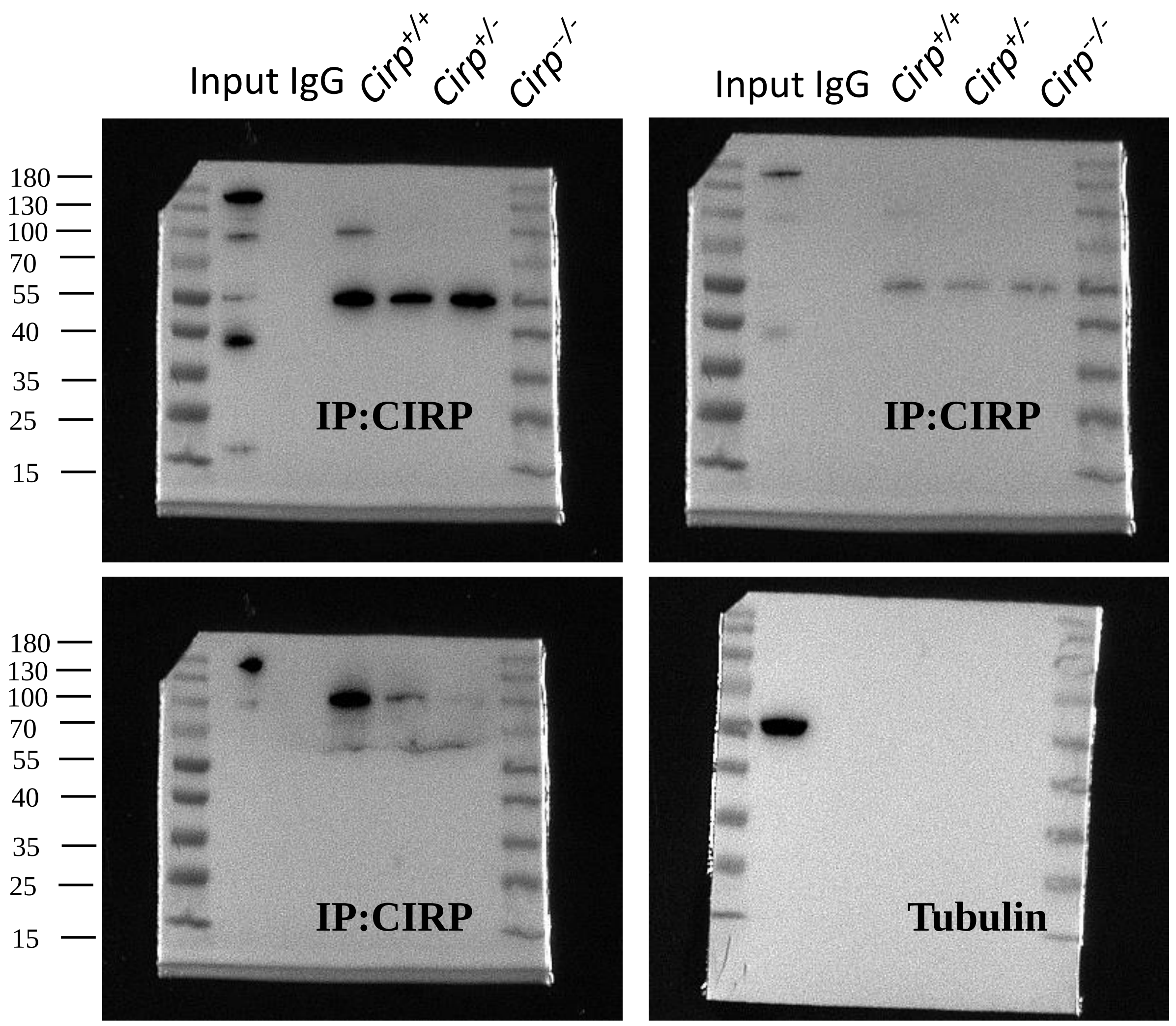

Fig8P

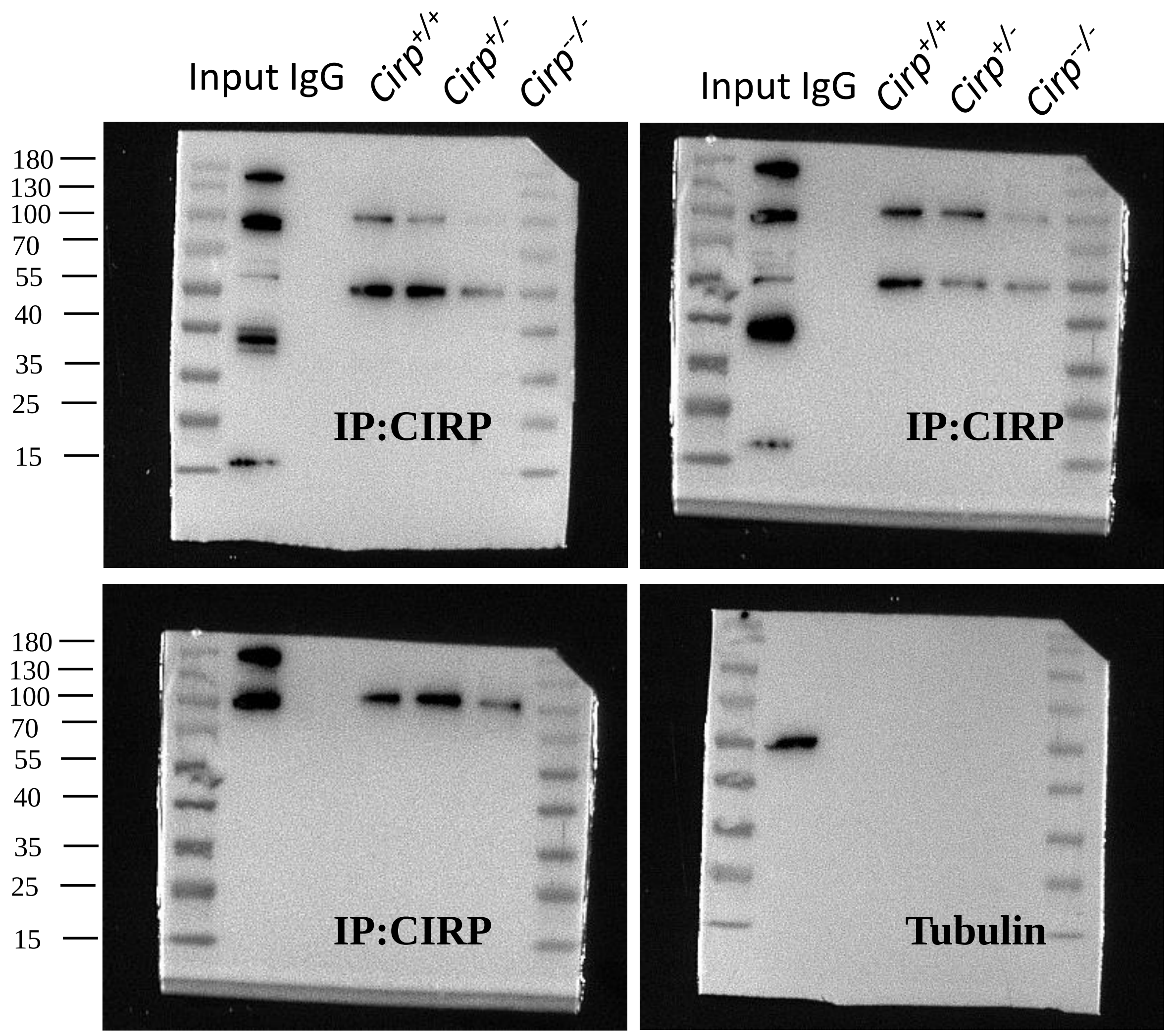

Western Blots in Figure 10

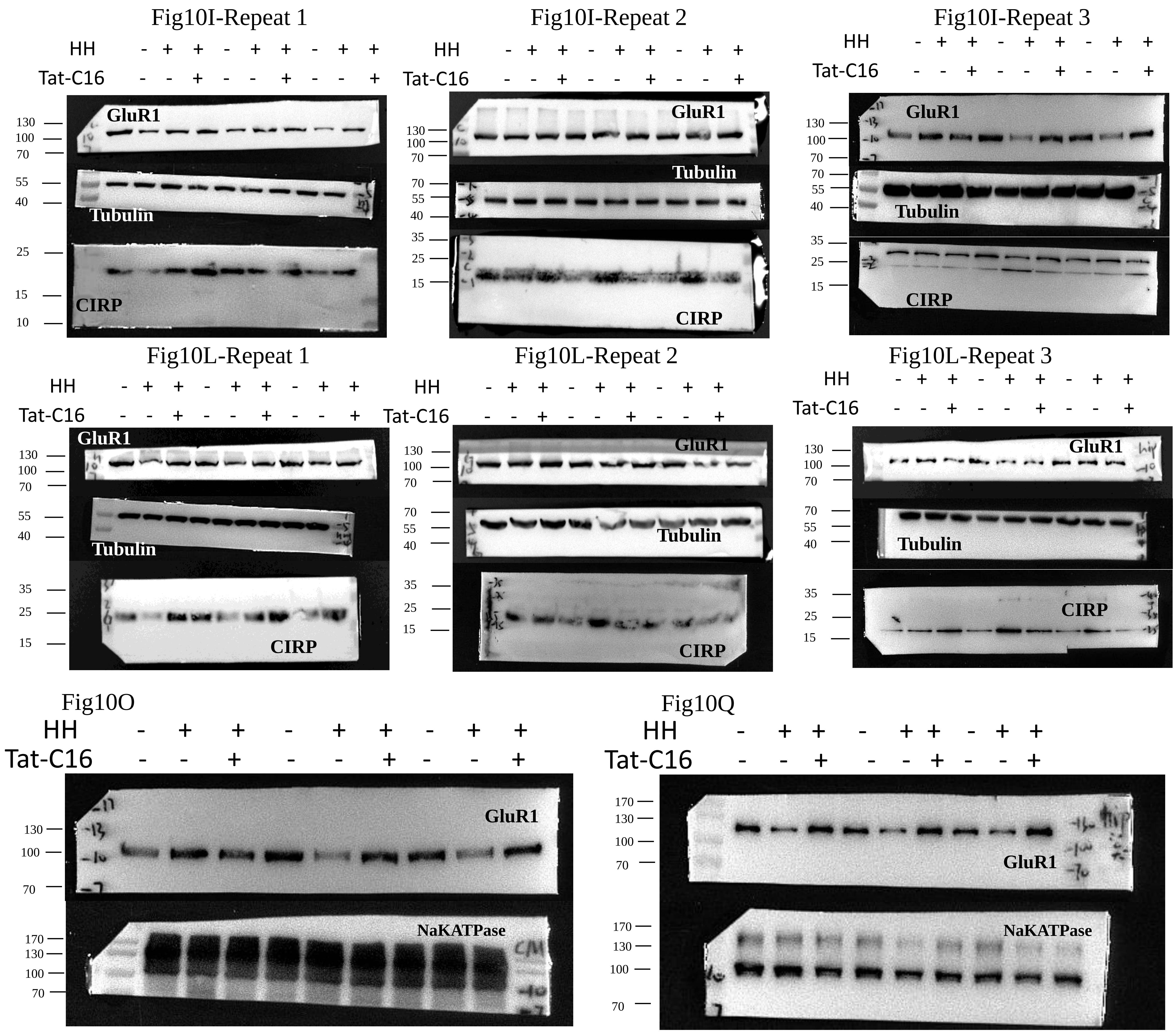

Western Blots in Figure 11

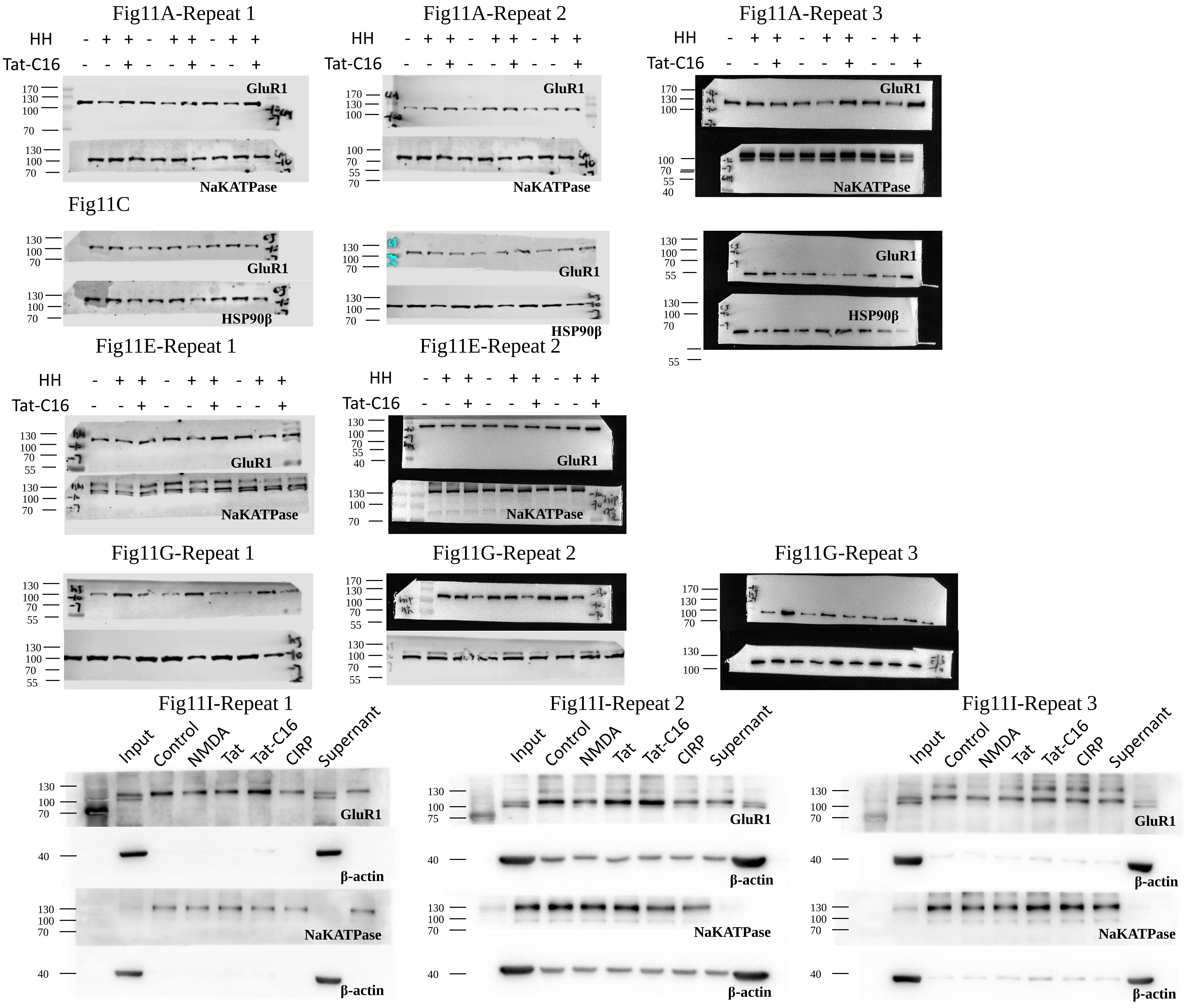

Western Blots in Figure 11

Fig11K

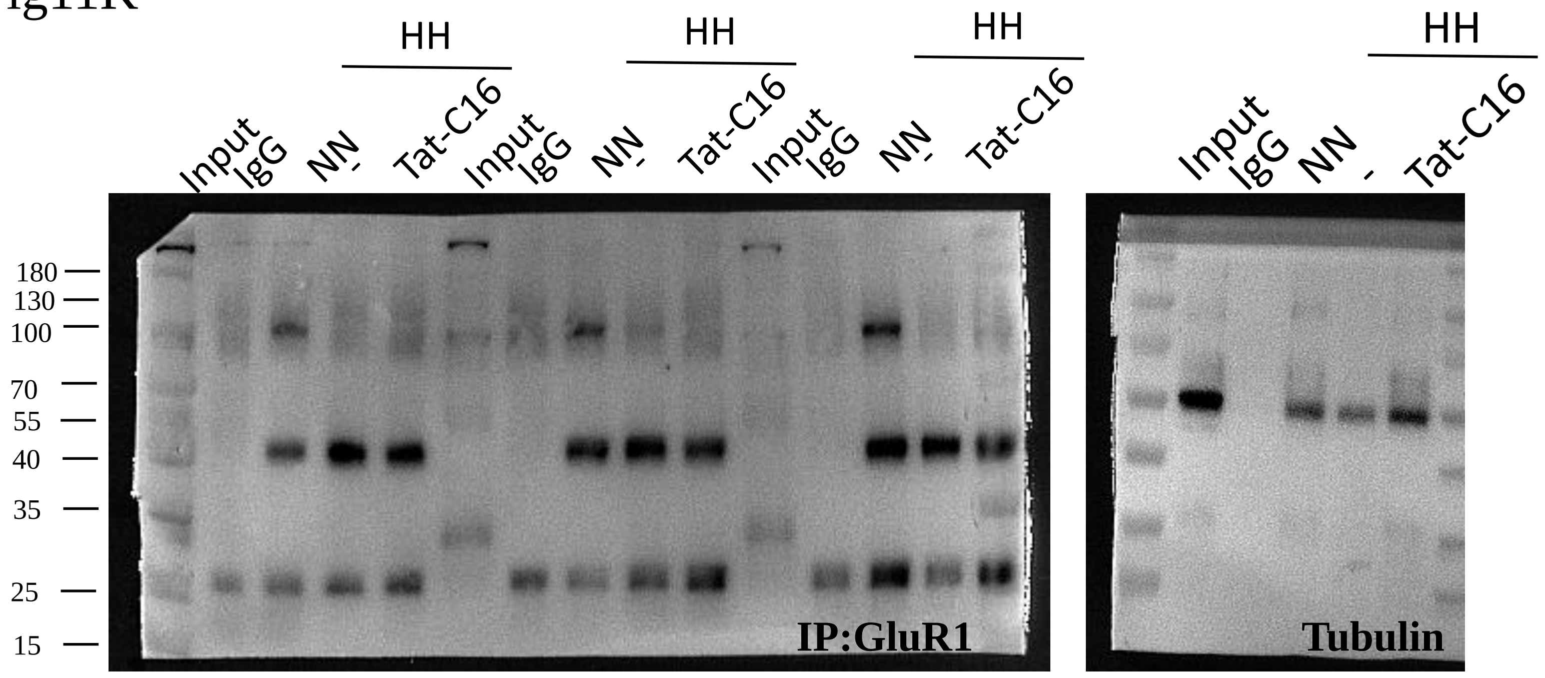

Fig11L

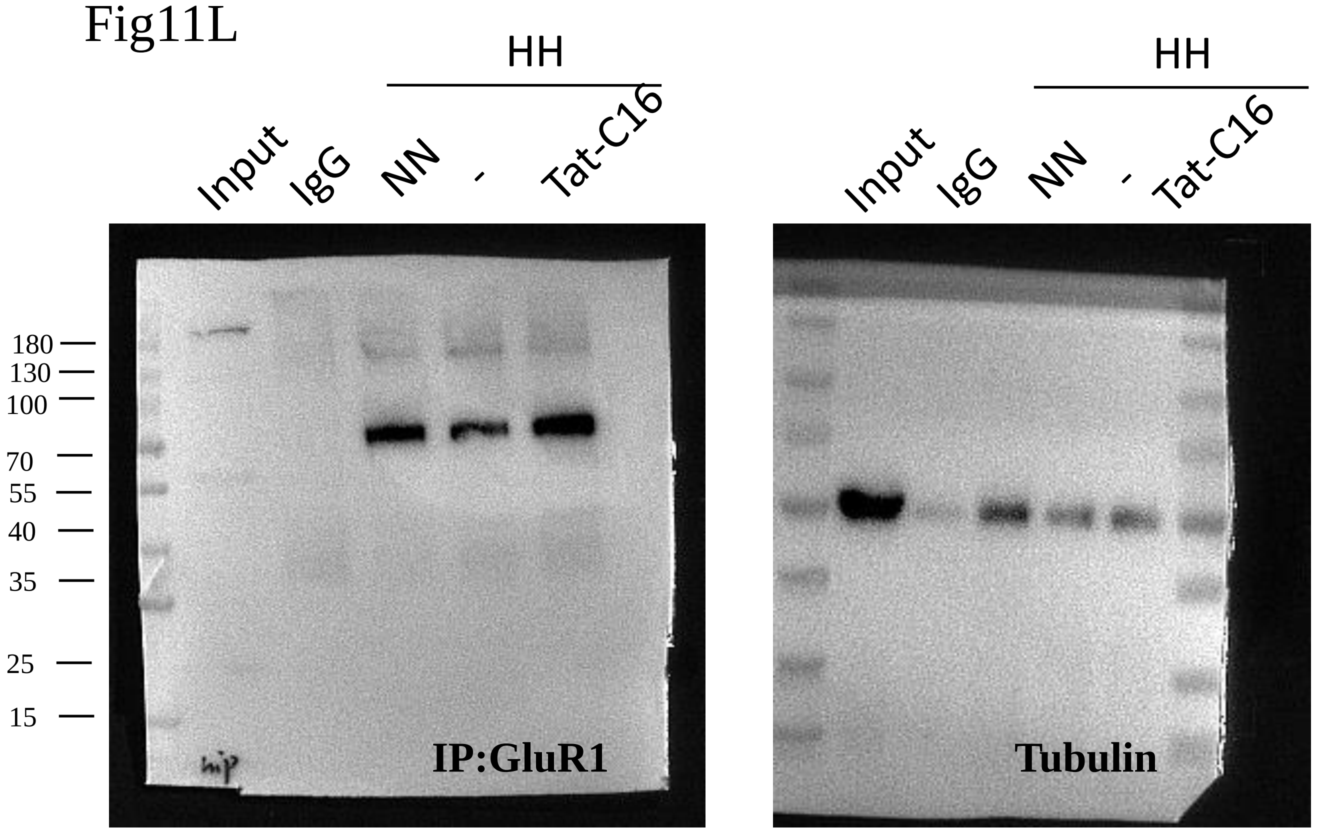

Fig11M

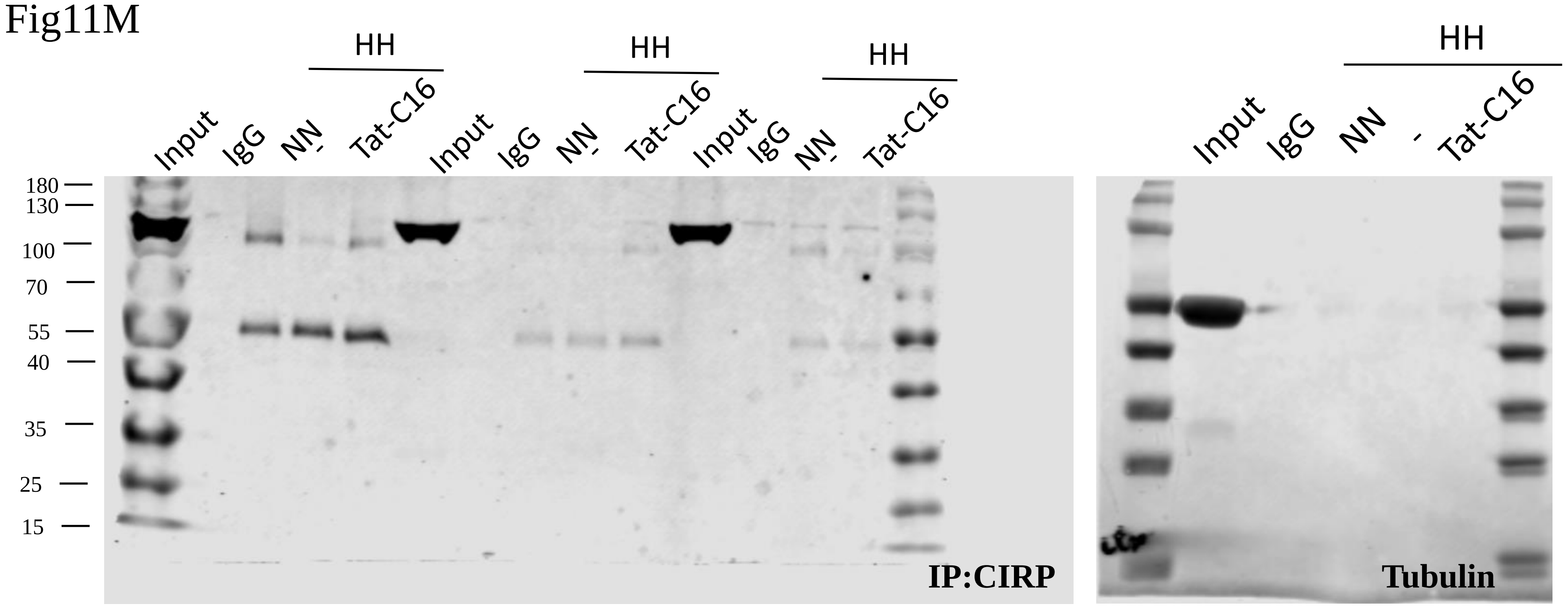

Fig11N

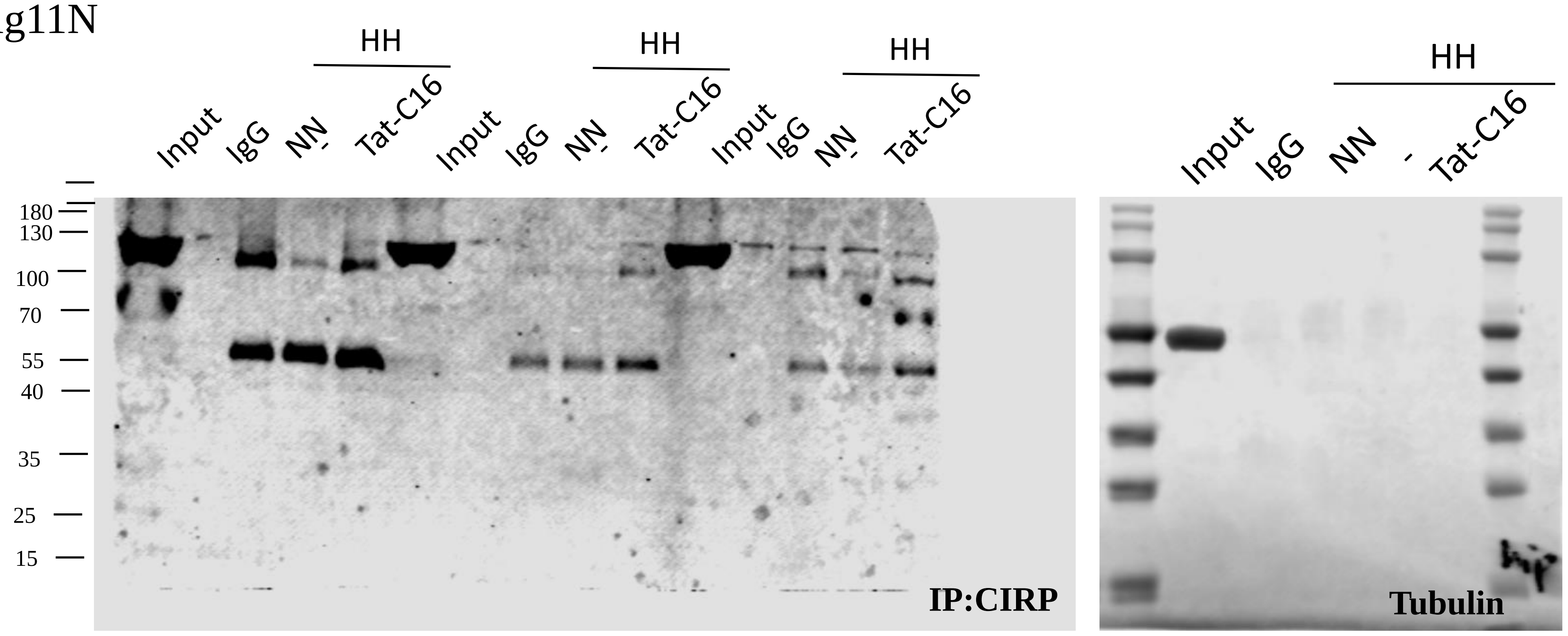

Western Blots in Supplementary Figure 1

Fig S1G

Cytomembrane

Hypoxia

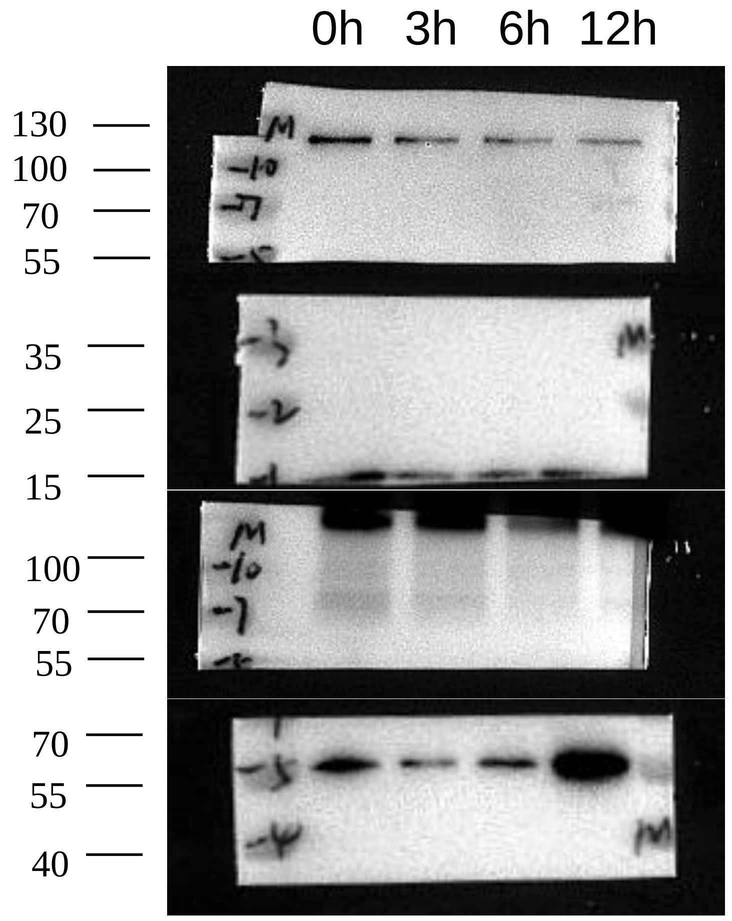

Fig S1H

Cytplasm

Hypoxia

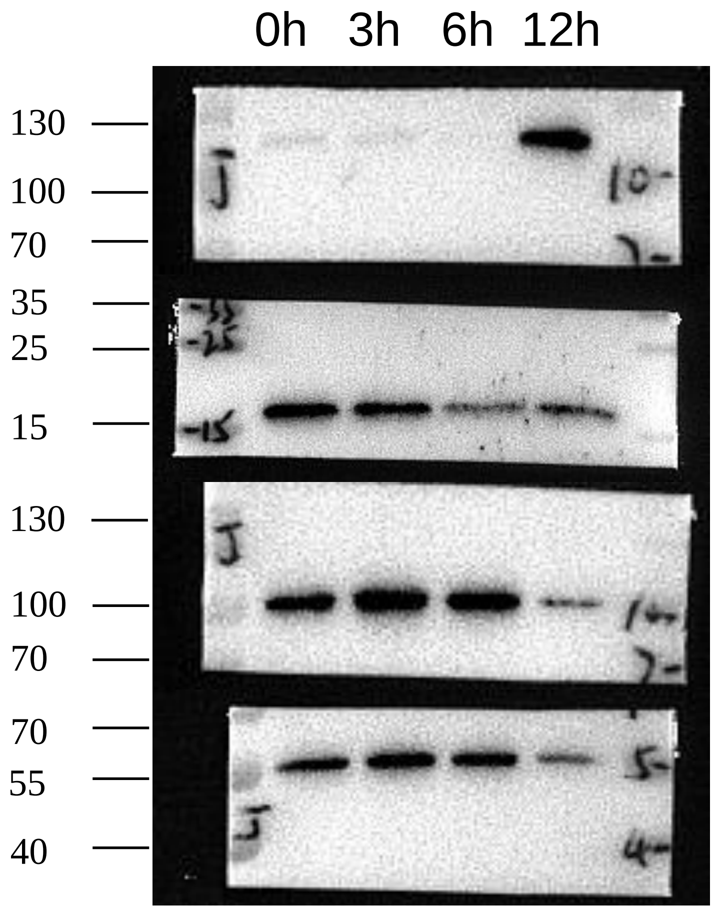

FigSE

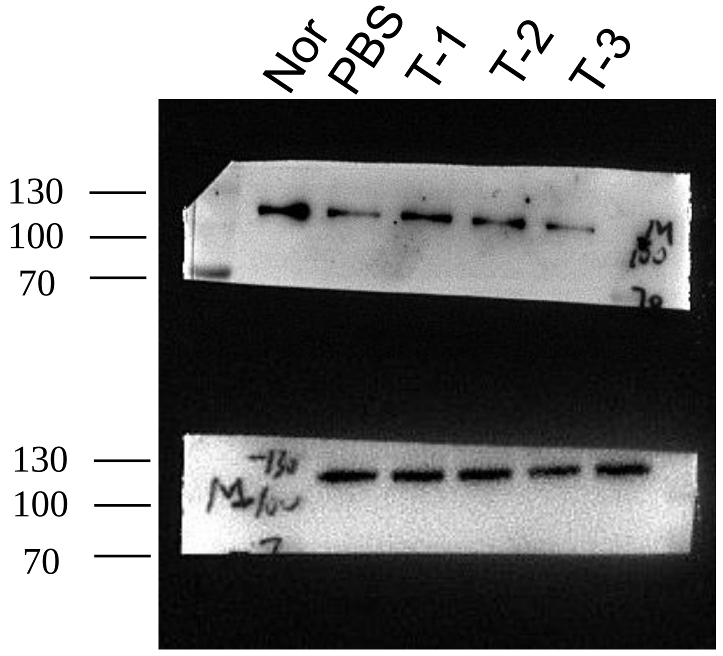

Supplement: Supplementary file 1 — Data S1. [file CNS-30-e70059-s004.pdf]
