# Supplementary figures and images for: Decreased cold‐inducible RNA‐binding protein (CIRP) binding to GluRl on neuronal membranes mediates memory impairment resulting from prolonged hypobaric hypoxia exposure
Source: CNS Neurosci Ther. 2024 Sep 24;30(9):e70059. doi: 10.1111/cns.70059 (PMC11420629; doi:10.1111/cns.70059)

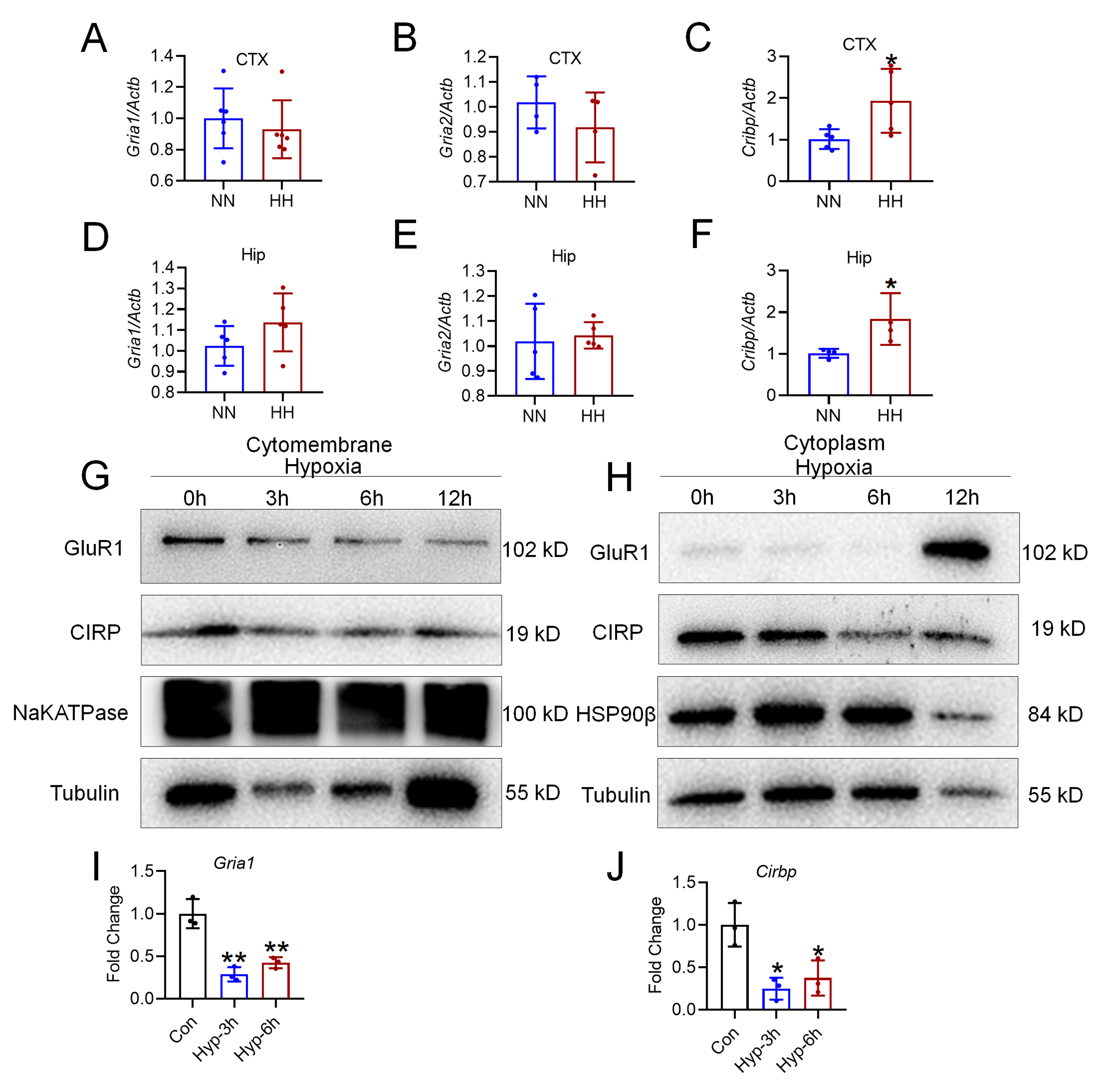

Supplement: Supplementary file 2 — Figure S1. [file CNS-30-e70059-s003.tif]

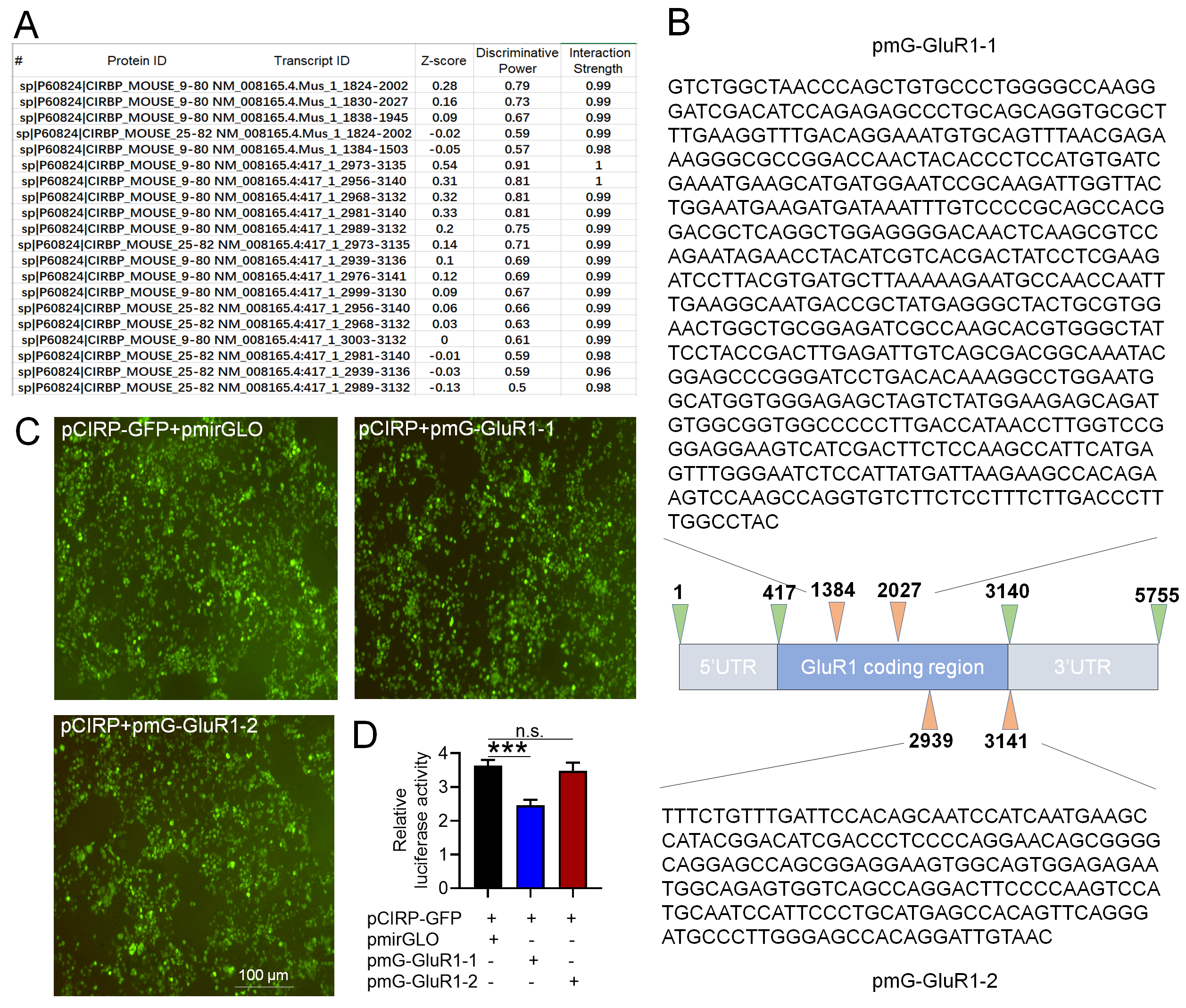

Supplement: Supplementary file 3 — Figure S2. [file CNS-30-e70059-s002.tif]
